# Effectiveness and Costs of Molecular Screening and Treatment for Bacterial Vaginosis to Prevent Preterm Birth: The AuTop Randomized Clinical Trial

**DOI:** 10.1001/jamapediatrics.2023.2250

**Published:** 2023-07-17

**Authors:** Florence Bretelle, Sandrine Loubière, Raoul Desbriere, Anderson Loundou, Julie Blanc, Hélène Heckenroth, Thomas Schmitz, Alexandra Benachi, Bassam Haddad, Franck Mauviel, Xavier Danoy, Pierre Mares, Nawal Chenni, Jean-Pierre Ménard, Jean-François Cocallemen, Nadia Slim, Marie Victoire Sénat, Céline Chauleur, Caroline Bohec, Gilles Kayem, Cynthia Trastour, André Bongain, Patrick Rozenberg, Valerie Serazin, Florence Fenollar

**Affiliations:** 1 Department of Obstetrics and Gynecology, La Conception Hospital, Assistance Publique–Hopitaux de Marseille, Marseille, France; 2 Aix-Marseille Univ, IRD, Assistance Publique–Hopitaux de Marseille, UMRD-258 Microbes, Evolution, Phylogenie and Infection (MEPHI), Marseille, France; 3 Research Unit EA 3279, CEReSS-Health Service Research and Quality of Life Center, Aix-Marseille University, Marseille, France; 4 Department of Obstetrics and Gynecology, Fondation Hopital Saint Joseph, Marseille, France; 5 Department of Obstetrics and Gynecology, Hopital Nord, Assistance Publique–Hopitaux de Marseille, Marseille, France; 6 Service de Gynécologie Obstétrique, Assistance Publique–Hôpitaux de Paris Hôpital Robert Debré, Université Paris Cité, Paris, France; 7 Service de Gynécologie-Obstétrique, DMU Santé des Femmes et des nouveau-nés Hôpital Antoine Béclère, Assistance Publique–Hôpitaux de Paris, Clamart, France; 8 Service de Gynécologie-Obstétrique, Hôpital Antoine Béclère, Assistance Publique–Hôpitaux de Paris, Université Paris Saclay, Clamart, France; 9 Centre Hospitalier de Créteil, Créteil, France; 10 Department of Obstetrics and Gynecology, Institut Mondor de Recherche Biomedicale, Université Paris Est Creteil, Centre Hospitalier Creteil, Creteil, France; 11 Department of Obstetrics and Gynecology, Centre hospitalier de Toulon sainte Musse, Toulon, France; 12 Departement of Obstetrics and Gynecology, Centre hospitalier d’Aix en Provence, Centre hospitalier de Pertuis, Aix en Provence, France; 13 Departement of Obstetrics and Gynecology, Centre hospitalier universitaire de Nimes, Nimes, France; 14 Departement of Obstetrics and Gynecology, Centre hospitalier d’Aubagne, Aubagne, France; 15 Direction de la Protection Maternelle et Infantile et de la Promotion de la Santé, Conseil départemental du Val-de-Marne, Créteil, France; 16 Departement de recherche clinique, Hopital Nord, Assistance hôpitaux de Marseille, Assistance Publique–Hopitaux de Marseille, Marseille, France; 17 Departement Gynécologie Obstétrique, Centre hospitalier Universitaire du Kremlin Bicetre, Kremlin Bicetre, France; 18 Clinical Epidemiology, Centre de Recherche en épidémiologie et Santé des populations, Paris Saclay University, Université de Versailles Saint-Quentin-en-Yvelines, Inserm, Team U1018, Villejuif, France; 19 Service de Gynécologie-obstétrique, CHU de Saint Etienne, INSERM, SAINBIOSE, U1059, Dysfonction Vasculaire et Hémostase, Université Jean-Monnet, Saint Etienne, France; 20 Centre Hospitalier de Pau, Pau, France; 21 Service de Gynécologie Obstétrique de l’hôpital Trousseau, Université Pierre et Marie Curie, INSERM U1153, Paris, France; 22 Departement d’Obstétrique-Reproduction-Gynécologie, Hôpital Archet, CHU de Nice, Nice, France; 23 American Hospital of Paris, Neuilly-sur-Seine, France; 24 Service de Biologie Médicale, CHI de Poissy-Saint-Germain-en-Laye, Poissy, France; 25 Université Paris-Saclay, Université de Versailles Saint-Quentin-en-Yvelines, Institut national de la recherche agronomique, Biologie de la Reproduction, Environnement, Epigénétique et Développement, Paris, France; 26 Department of Infectious Diseases, Hopital de la Timone, Assistance Publique–Hopitaux de Marseille, IHU-Méditerranée Infection, Marseille, France; 27 Aix-Marseille Univ, Institut recherche et développement, Assistance Publique–Hopitaux de Marseille, SSA, Vecteurs – Infections Tropicales et Méditeranéennes, Marseille, France

## Abstract

**Importance:**

Bacterial vaginosis (BV) is a well-known risk factor for preterm birth. Molecular diagnosis of BV is now available. Its impact in the screening and treatment of BV during pregnancy on preterm births has not been evaluated to date.

**Objective:**

To evaluate the clinical and economic effects of point-of-care quantitative real-time polymerase chain reaction screen and treat for BV in low-risk pregnant women on preterm birth.

**Design, Setting, and Participants:**

The AuTop trial was a prospective, multicenter, parallel, individually randomized, open-label, superiority trial conducted in 19 French perinatal centers between March 9, 2015, and December 18, 2017. Low-risk pregnant women before 20 weeks’ gestation without previous preterm births or late miscarriages were enrolled. Data were analyzed from October 2021 to November 2022.

**Interventions:**

Participants were randomized 1:1 to BV screen and treat using self-collected vaginal swabs (n = 3333) or usual care (n = 3338). BV was defined as *Atopobium vaginae* (*Fannyhessea vaginae*) load of 10^8^ copies/mL or greater and/or *Gardnerella vaginalis* load of 10^9^ copies/mL or greater, using point-of-care quantitative real-time polymerase chain reaction assays. The control group received usual care with no screening of BV.

**Main Outcomes and Measures:**

Overall rate of preterm birth before 37 weeks’ gestation and total costs were calculated in both groups. Secondary outcomes were related to treatment success as well as maternal and neonate health. Post hoc subgroup analyses were conducted.

**Results:**

Among 6671 randomized women (mean [SD] age, 30.6 [5.0] years; mean [SD] gestational age, 15.5 [2.8] weeks), the intention-to-treat analysis of the primary clinical and economic outcomes showed no evidence of a reduction in the rate of preterm birth and total costs with the screen and treat strategy compared with usual care. The rate of preterm birth was 3.8% (127 of 3333) in the screen and treat group and 4.6% (153 of 3338) in the control group (risk ratio [RR], 0.83; 95% CI, 0.66-1.05; *P* = .12). On average, the cost of the intervention was €203.6 (US $218.0) per participant, and the total average cost was €3344.3 (US $3580.5) in the screen and treat group vs €3272.9 (US $3504.1) in the control group, with no significant differences being observed. In the subgroup of nulliparous women (n = 3438), screen and treat was significantly more effective than usual care (RR, 0.62; 95% CI, 0.45-0.84; *P* for interaction = .003), whereas no statistical difference was found in multiparous (RR, 1.30; 95% CI, 0.90-1.87).

**Conclusion and Relevance:**

In this clinical trial of pregnant women at low risk of preterm birth, molecular screening and treatment for BV based on *A vaginae* (*F vaginae*) and/or *G vaginalis* quantification did not significantly reduce preterm birth rates. Post hoc analysis suggests a benefit of screen and treat in low-risk nulliparous women, warranting further evaluation in this group.

**Trial Registration:**

ClinicalTrials.gov Identifier: NCT02288832

## Key Points

**Question:**

What are the medical and economic effects of screen and treat for bacterial vaginosis using point-of-care quantitative real-time polymerase chain reaction during pregnancy?

**Findings:**

In this randomized clinical trial including 6671 pregnant women enrolled before 20 weeks’ gestation assigned to screen and treat or usual care, the preterm birth rate was 3.8% and 4.6%, respectively, which was not significantly different. Total costs were also not significantly different.

**Meaning:**

Compared with usual care, screen and treat for bacterial vaginosis did not reduce the risk of preterm birth; however, this strategy should be further evaluated in nulliparous and high-risk multiparous women.

## Teaser

This randomized clinical trial evaluates the clinical and economic effects of point-of-care quantitative real-time polymerase chain reaction screen and treat for bacterial vaginosis in low-risk pregnant women on preterm birth.

## Introduction

Preterm birth affects approximately 5% to 11% of births worldwide, with variation by country, ethnicity, or other factors. This figure has remained constant despite different preventive strategies or treatments.^1^ Among the risk factors, bacterial vaginosis (BV) is well known.^2^ BV is a common vaginal dysbiosis, with a predominance of anaerobic bacteria associated with a lack of lactobacillus, detected with various diagnosis methods. Often asymptomatic, BV increases the risk of preterm birth from 2-fold to 7-fold according to the gestational age at diagnosis; the earlier the age, the higher the risk.^3^ The conventional diagnosis of BV can be clinical according to Amsel criteria or based on Nugent criteria, vaginal pH, or molecular diagnosis.^4,5,6^

Debate remains regarding the effectiveness of screening and treating BV during pregnancy.^7,8,9^ Three meta-analyses evaluated the impact on pregnancy outcome of screen and treat interventions for pregnant women using conventional diagnosis methods for BV.^7,10,11^ Lamont et al^11^ (5 studies and 2346 patients) reported a benefit of screen and treat using clindamycin. The Cochrane Database update (21 studies and 7847 patients) does not recommend screening for BV^7^ but highlights that 2 studies that included intermediate flora as part of their criteria showed a 50% reduction in preterm delivery and an 80% reduction in miscarriages.^12,13^ More recently and after the implementation of the present study, the updated systematic review for US Preventive Services Task Force^10^ (n = 48 studies), including the results from the large PREMEVA study,^14^ still showed that conventional screening tests for BV varied in accuracy, suggested no efficacy of treatment for asymptomatic BV in a general obstetric population, and was inconclusive for women with a history of prior preterm delivery. Based on this literature, international and French recommendations advise against screening with conventional diagnosis tools in low-risk populations,^13,14,15^ even though 65% of preterm births occur in patients with no obstetrical history or identified risk factors.^16^

Recently, molecular biology has been shown to more accurately identify vaginal microbiota than other methods.^17,18^ For example, molecular biology showed that among women with a Nugent score greater than 4 (intermediate vaginal flora), 57% had true BV,^19^ suggesting that 43% of those women did not have BV. Conversely, molecular tools provide an objective, reproducible, quantitative diagnosis of BV.^6,19^ It identifies emergent pathogen species with fastidious culture, such as *Atopobium vaginae* (recently renamed *Fannyhessea vaginae*). To our knowledge, Fredrick et al^17^ provide one of the first studies of vaginal microbiota and its link with pregnancy outcomes. Our team has previously reported that *A vaginae* and *Gardnerella vaginalis* vaginal loads are associated with preterm birth and shortened length of pregnancy in case of threatened preterm labor.^20,21^

Differences in the choice of treatments represent another major factor in the difficulty in interpretation of previous studies. There are international and national recommendations for the use of metronidazole or clindamycin for at high risk of preterm delivery pregnancies with a diagnosis of BV.^22,23^ The National Institute for Health and Care Excellence recommend that either oral or vaginal antibiotics be considered (ie, metronidazole, clindamycin, or amoxicillin).^24^ Thus, literature does not allow to choose one treatment over another.^7^ Overall, the discrepancies in meta-analyses depict the difficulty of reaching definitive conclusions about screening and treatment efficacy and point out the need for further studies, in particular using new tools like molecular testing. To our knowledge, there are no randomized studies to date evaluating the impact of screen and treat strategies using molecular biology during pregnancy, except for 2 ongoing studies.^25,26^ Therefore, we conducted a prospective multicenter randomized clinical trial based on molecular standardized BV diagnosis before 20 weeks’ gestation with control of vaginal swabs after treatment in a low-risk population to determine whether the intervention is cost-effective in reducing the rate of preterm birth.

## Methods

### Ethics Compliance

The study was approved by the South Mediterranean Committee for the Protection of Research Subjects and the French National Agency of Medicine and Health Products Safety. All patients gave written informed consent.

### Trial Design

This study was a multicenter individually randomized open-label superiority trial conducted in a low-risk population of pregnant women. The study was conducted in 19 French maternity hospitals. The AuTop protocol was published,^27^ and a detailed version is provided in Supplement 1. The statistical analysis plan can be found in Supplement 2.

### Participants

The study targeted pregnant women in early pregnancy. Inclusion criteria were pregnant women 18 years and older before 20 weeks’ gestation, regardless of their parity, with no history of preterm birth or late abortion and with no major risk factors for prematurity, including absence of diabetes, systemic lupus erythematosus, treated hypertension, fetal malformation, cervical conization, or multiple pregnancy. Patients were excluded at the time of enrollment if they were deprived of their freedom by a court or administrative decision, were under legal protection, had an extra-uterine pregnancy or nonprogressive pregnancy, had received antibiotic treatment in the week prior to inclusion, or were participating in another biomedical research protocol. Women who did not understand written and spoken French were also excluded. Race and ethnicity data were collected by self-report.

### Randomization

After providing informed consent, women were randomly assigned, using a web-based system with a 1:1 ratio, to either undergo molecular screening and treatment of BV using self-collected vaginal swabs (screen and treat group) or receive no screening (control group). The randomization list used a permuted block-design (block size of 6) and was stratified by center. The electronic case report form was developed using the online system CleanWeb.^28^ Study participants and health professionals, including gynecologists and midwives, were unblinded due to the nature of the procedure. The statistician and health economist were masked to study group until the data were analyzed.

### Intervention

In the intervention group, women underwent systematic screening for BV via analysis of their self-collected vaginal samples. In case of BV detection, a treatment was prescribed within a maximum of 24 to 48 hours after detection. Treatment consisted of azithromycin, 1 g, repeated after 48 hours, or 2 g of amoxicillin per day for 7 days. Women with BV returned vaginal control self-swabs after 15 days and each month until 28 weeks’ gestation. Each patient with a positive test result had a total of 4 self-collected vaginal samples (eFigure in Supplement 3). No probiotics were given during the study.

### Molecular Diagnosis of BV

The investigators have previously developed a molecular biology–based rapid diagnostic tool, applicable as a point-of-care testing strategy, for the diagnosis of BV using specific quantitative real-time polymerase chain reaction (qPCR) assays to quantify the DNA levels of *A vaginae* and *G vaginalis*.^19^ The sequences of the primers and probes used are detailed in eTable 1 in Supplement 3, and the molecular analysis procedure in eMethods 1 in Supplement 3. This tool has been patented (European Patent Office No. 2087134; eMethods 2 in Supplement 3). Compared with the reference techniques, the tool has reported a higher specificity (99%), sensitivity (95%), and positive (95%) and negative (99%) predictive values.^5,19^ The point-of-care test was considered positive if *A vaginae* was detected at a threshold of more than 10^5^ DNA copies/mL and/or *G vaginalis* at a threshold of more than 10^5^ DNA copies/mL, and BV was defined as *A vaginae* of 10^8^ DNA copies/mL or more and/or a *G vaginalis* of 10^9^ copies/mL or more, based on previous studies.^19,21^

The control group received usual care according to the standard practices with no systematic screening of BV. Health professionals were free to prescribe a standard vaginal swab if symptoms were present.

### Data Sources

Data were collected from 2 sources: clinical and resource use data were obtained from hospital administrative databases, and data on treatment adverse effects and outpatient care were gathered from patient self-reports during telephone interviews at the end of each treatment session or during an interview at hospital discharge. Unit costs were estimated using data from the French National Hospital Database, the French Register of Pharmaceutical Specialties, and national tariffs. All resources were valued in 2019 euros.

### Primary Outcomes

Clinical and economic outcomes were compared between groups, and if found relevant, an incremental cost-effectiveness ratio, expressed as the incremental cost per additional unit of effectiveness gained, was calculated. The effectiveness outcome was the rate of births before 37 weeks’ gestation. Total costs included screening with point-of-care qPCR, control vaginal swabs for women with positive test results and subsequent antibiotic treatments, antenatal hospital admissions, physicians’ consultations, management of complications during pregnancy (either through inpatient or outpatient care), and neonatal care for full-term and preterm infants.

### Secondary and Exploratory Outcomes

Prespecified secondary clinical outcomes were rates of preterm birth before 26, 28, and 32 weeks’ gestation, premature rupture of membranes, intrauterine growth restriction, endometritis, and total hospital length of stay. Other exploratory outcomes measured in the intervention group were rate of BV, treatment recurrence rate (defined as a positive control vaginal swab using qPCR after a previous control vaginal swab was negative), spontaneous abortion (before 22 weeks’ gestation), late miscarriage (between 22 and 24 weeks’ gestation), fetal death, preeclampsia, vaginal bleeding, neonatal infection, transfers, length of stay, and neonatal mortality.

### Sample Size

The control group was expected to have a preterm birth rate of 4.3% and the screen and treat group a preterm birth rate of 3.0%.^9^ With a statistical power of 80%, a threshold for statistical significance set at a *P* value of .05, and assuming that 20% of patients will be lost to follow-up, the sample size was calculated to be 6800 women (3400 per group) to achieve statistical significance of the effect size. This sample size enabled the cost-effectiveness of the screen and treat intervention to be assessed at an estimated threshold of €22 500 (US $24 089) with an expected incremental cost of €230 (US $246).

### Statistical Analysis

In the primary analysis, the intention-to-treat population was considered, including all patients who were randomized and had provided at least baseline characteristics. Analyses followed a statistical analysis plan that was written before data collection was completed and analysis began (Supplement 2). Missing data were handled using multiple imputations.^29^ Imputed data sets were implemented using multivariate imputation by chained equations (MICE) and mitools R packages. Missing data regarding the primary medical outcome (4.5%) were addressed using multiple imputations.^29^

Primary and secondary outcomes were compared between the 2 groups using *t* test or Mann-Whitney *U* test for continuous variables and χ^2^ or Fisher exact tests for categorical variables. Risk ratios (RRs) with 95% CIs were estimated.

A post hoc subgroup analysis was assessed for the primary outcome as follows. RRs were considered with tests for the interactions between the study group and prior identified subgroups. Three subgroups were examined: women older than 30 years, tobacco users, and nulliparous women. Imputation models were implemented using the MICE and miceadds R packages (R studio version 3.6.0; The R Foundation), with statistical significance set at 2-sided *P* < .05.

## Results

**Figure 1.**
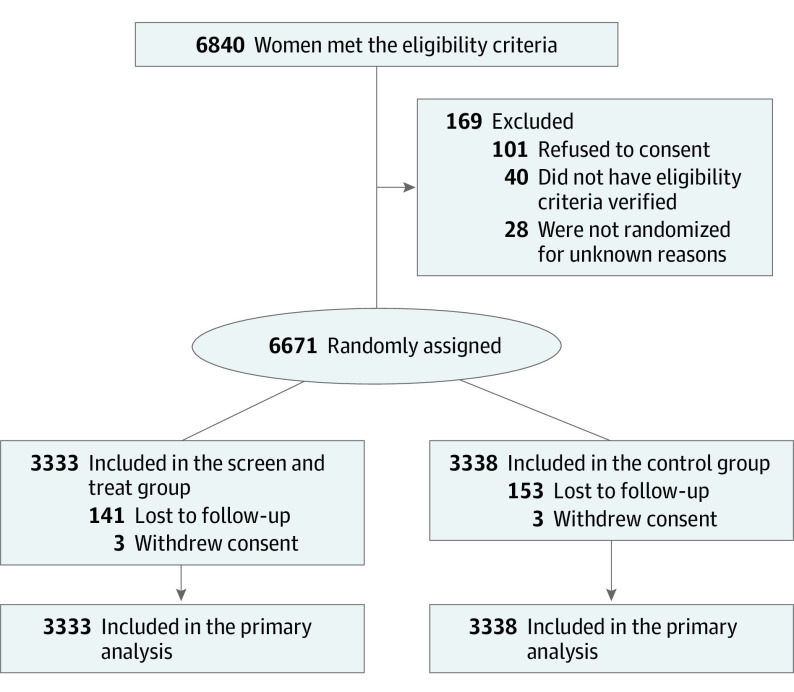
Flow of Participants in the AuTop Trial

Between March 9, 2015, and December 18, 2017, 6671 patients (mean [SD] age, 30.6 [5.0] years; mean [SD] gestational age, 15.5 [2.8] weeks) were randomly assigned to study groups: 3333 to the screen and treat group and 3338 to the control group (Figure 1). Demographic characteristics and medical history were similar in the 2 groups (Table 1). At inclusion, 1671 patients (50%) in the screen and treat group and 1767 (52%) in the control group were nulliparous. At the end of the study follow-up on November 18, 2019, the median (range) duration of follow-up was 24 (13-117) weeks. Outcomes of pregnancy were known for all but 300 of the randomized patients, including 143 (4.3%) in the screen and treat group and 157 (4.7%) in the control group.

**Table 1.** Characteristics of the Participants According to Randomly Assigned Group

Characteristic	No. (%)	
	Screen and treat (n = 3333)	Control (n = 3338)
Age, mean (SD), y	30.5 (5.0)	30.7 (5.1)
Racial and ethnic group^a^		
Asian	57 (1.8)	55 (1.7)
Caucasian^b^	147 (4.5)	144 (4.3)
European	1968 (60.7)	1982 (61.5)
North African	762 (23.5)	725 (21.9)
Sub-Saharan African	306 (9.4)	317 (9.6)
Other race or ethnicity	77 (2.3)	89 (2.7)
Bachelor’s degree or higher education^a^	2716 (82.1)	2761 (83.3)
Employed^a^	2312 (69.8)	2329 (70.1)
Nulliparous	1671 (50.1)	1767 (52.8)
Previous early miscarriage^c^	737 (22.2)	742 (22.3)
Previous abortion	633 (19.0)	645 (19.4)
Gestational age at inclusion, mean (SD), wk	15.5 (2.8)	15.5 (2.8)
Assisted reproductive technology use	189 (5.7)	203 (6.1)
Body mass index, mean (SD)^d^	24.2 (4.9)	24.0 (4.7)
Tobacco use^a^	364 (11.0)	361 (10.9)
Smoking during pregnancy (>10 cigarette/d)^a^	20 (0.6)	24 (0.7)
Alcohol use^a^	29 (0.9)	33 (1.0)
Vaginal toilet practice during pregnancy^a^	309 (9.4)	305 (9.3)

^a^ Variable was self-reported.

^b^ Native to the Caucasus region.

^c^ Previous abortion includes therapeutic and spontaneous abortions.

^d^ Calculated as weight in kilograms divided by height in meters squared.

### Screening and Treatment Outcomes

The screening and treatment outcomes are described in Table 2. In the screen and treat group, 242 (7.3%) had BV. Among the patients with BV, 44 (18.2%) did not receive treatment. The initial success rate and recurrence rate were 46.8% (44 of 198) and 32.6% (30 of 92), respectively.

**Table 2.** Screening and Treatment Outcomes

Outcome	No. (%)		
	Screen and treat	Nulliparous women	Multiparous women
Screening outcomes			
Total, No.	3333	1671	1662
Molecular diagnosis done	3329 (99.9)	1668 (99.8)	1660 (99.9)
*Atopobium vaginae* (*Fannyhessea vaginae*) load >10.8 copies/mL	185 (5.6)	91 (5.5)	94 (5.7)
*Gardnerella vaginalis* load >10^9^ copies/mL	123 (3.7)	58 (3.5)	65 (3.9)
Bacterial vaginosis	242 (7.3)	113 (6.8)	129 (7.8)
Treatment outcomes			
Total, No.	242	113	129
Treatment prescribed	198 (81.8)	96 (85.0)	102 (79.1)
Initial success	92 (46.8)	50 (52.0)	43 (42.1)
Related recurrence	30 (32.6)	12 (24.0)	18 (42.8)
Total recurrences after 4 swabs	54 (27.3)	22 (22.9)	32 (31.7)
Total failures after 4 swabs	44 (22.2)	9 (9.4)	15 (14.7)

### Primary Outcome

The intention-to-treat analysis of the primary clinical outcome showed no evidence of a reduction in the rate of preterm birth with the screen and treat strategy compared with usual care. The rate of preterm birth was 3.8% (127 of 3333) among women in the screen and treat and 4.6% (153 of 3338) among women in the control group (RR, 0.83; 95% CI, 0.66-1.05; *P* = .12) (Table 3). Sensitivity analyses with complete cases and with missing values imputed using the worst-case scenario yielded similar results (eTable 3 in Supplement 3).

**Table 3.** Primary, Secondary, and Exploratory Outcomes According to Randomly Assigned Groups

Outcome	No. (%)		RR (95% CI)	*P* value
	Screen and treat (n = 3333)	Control (n = 3338)		
Primary outcomes				
Birth before 37 weeks’ gestation	127 (3.8)	153 (4.6)	0.83 (0.66-1.05)	.12
Total costs, mean (SD), € [US $]	3344.3 (2562.9) [3605.2]	3272.9 (3637.8) [3528.2]	0.96 (0.95-1.01)	.23
Secondary and exploratory outcomes				
Gestational age, mean (SD)	37.58 (2.55)	37.52 (2.51)	1.00 (0.99-1.01)	.37
<26 wk	6 (0.2)	9 (0.3)	0.66 (0.24-1.82)	.44
<28 wk	8 (0.2)	11 (0.3)	0.72 (0.29-1.77)	.50
<32 wk	26 (0.8)	34 (1.0)	0.76 (0.46-1.26)	.31
In subgroups				
Nulliparous, mean (SD)	37.67 (2.43)	37.48 (2.58)	1.01 (1.00-1.02)	.03
Multiparous, mean (SD)	37.49 (2.67)	37.57 (2.42)	0.99 (0.98-1.01)	.36
Other pregnancy ending				
Spontaneous abortion (<22 weeks’ gestation)	13 (0.4)	10 (0.3)	1.30 (0.58-2.91)	.59
Fetal death	20 (0.6)	12 (0.4)	1.65 (0.82-3.34)	.18
Medical abortion/late miscarriage	13 (0.6)	19 (0.4)	0.74 (0.37-1.48)	.39
Pregnancy complications				
Premature rupture of membranes	39 (1.2)	55 (1.6)	0.71 (0.47-1.06)	.10
Intrauterine growth restriction	72 (2.1)	65 (2.0)	1.11 (0.80-1.55)	.70
Preeclampsia	48 (1.4)	47 (1.4)	1.01 (0.68-1.50)	.88
Vaginal bleeding	84 (2.5)	66 (2.0)	1.27 (0.92-1.74)	.10
Endometritis	5 (0.2)	10 (0.3)	0.75 (0.52-1.07)	.30
Length of stay for delivery, median (IQR), d	4.0 (3.0-5.0)	4.0 (3.0-5.0)	0.97 (0.95-1.01)	.96
Neonatal outcomes				
Birth weight, median (IQR), g	3313.0 (3015.3-3624.8)	3302.3 (2997.0-3636.1)	1.01 (0.99-1.01)	.96
Birth weight <2500 g	152 (4.6)	157 (4.7)	0.96 (0.77-1.20)	.73
Apgar score <7 at 5 min	45 (1.4)	30 (0.9)	1.53 (0.96-2.42)	.08
NICU care	41 (1.2)	50 (1.5)	0.83 (0.55-1.25)	.37
Transfer	120 (3.6)	139 (4.1)	0.87 (0.68-1.10)	.36
Neonate death	4 (0.1)	3 (0.1)	1.31 (0.31-5.62)	.71
Length of stay, median (IQR), d	3.0 (3.0-4.0)	3.0 (3.0-4.0)	0.94 (0.92-1.01)	.85

Abbreviations: NICU, neonatal intensive care unit; RR, risk ratio.

On average, the cost of the intervention was €203.6 (US $218.0) per woman, and the total average cost was €3344.3 (US $3580.5) in the screen and treat group vs €3272.9 (US $3504.1) in the control group, with no significant differences being observed. Details of mean costs are provided in eTable 2 in Supplement 3.

### Secondary and Exploratory Outcomes

With respect to the 19 secondary end points, we noted no evidence of the statistically superiority of screen and treat strategy over usual care (Table 3). Gestational age at delivery, endometritis rate, and other complications rates during pregnancy did not differ between groups. There was no difference in the length of hospital stay for mothers or newborns (median [IQR] of 4 [3-5] days and 3 [3-4] days, respectively) between groups. Newborn morbidity and mortality were not different between groups.

### Subgroup Analysis

**Figure 2.**
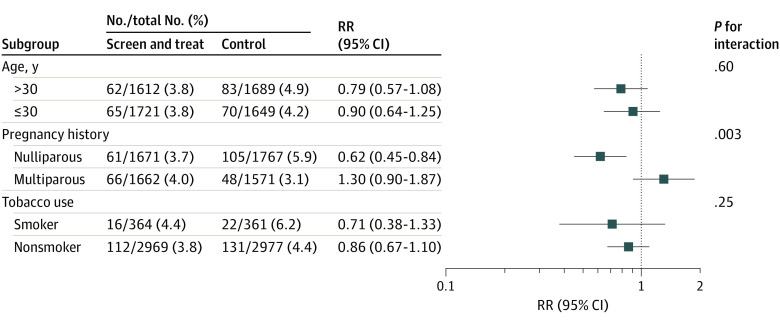
Subgroup Analyses of the Primary Outcome *P* values for interaction were obtained from the interactions between the study group and the variable which identified the subgroup. RR indicates risk ratio.

Characteristics were compared in the subgroups of nulliparous and multiparous women according to randomly assigned groups, with nonsignificant differences found (eTable 4 in Supplement 3). Associations between treatment and preterm births across the subgroups are summarized in Figure 2. With a statistically significant interaction term, the screen and treat effect varied according to whether the women were nulliparous (RR, 0.62; 95% CI, 0.45-0.84) or multiparous (RR, 1.30; 95% CI, 0.90-1.87; *P* for interaction = .003). Among nulliparous women, the number of preterm births was significantly lower in the screen and treat than in the control group (61 of 1671 [3.6%; 95% CI, 2.9-4.6] vs 105 of 1767 [5.9%; 95% CI, 4.8-7.2]), at a nonsignificantly lower total cost (€3632.4 [US $3888.9] vs €3715.9 [US $3978.3]; *P* = .33). There was no statistical difference between preterm rate and treatment groups in the other subgroup analyses.

## Discussion

To our knowledge, our study is the largest prospective randomized study including low-risk pregnant women and the first to use molecular tools for the diagnosis of BV. Our intervention based on a molecular screening and treatment of positive BV did not significantly reduce the relative risks of preterm birth or improve secondary maternal and neonate outcomes. In contrast to the apparent lack of benefit in the overall study population, in the subgroup of nulliparous women, the effect of the intervention was a significant 38% reduction in risk of preterm births (RR, 0.62; 95% CI, 0.45-0.84). To note, among the 6671 study participants, more than 3438 women were nulliparous, and were distributed in a balanced way between the 2 groups. The lower risk of preterm birth in nulliparous women could be explained by the fact that these women had an unknown risk of preterm birth early in pregnancy. Conversely, multiparous women in our study had a very low risk because they had no history of preterm birth or late miscarriage. The lower treatment rate in multiparous women (79.1% [102 of 129] vs 85.0% [96 of 113]) may be another explanation. Because the literature reports differences in the level of risk factors for preterm birth in multiparous and nulliparous women, we can speculate that the effect of treatment would be different in these subgroups.^30,31^ In the AuTop trial, women included in the screen and treat group were enrolled in a screening program. Thus, the risk perception of the women and their obstetricians/midwives could have been modified. This may partly explain our results in favor of the intervention.

The strengths of our study lie in its design and methodology. First, randomization ensured that demographic characteristics and baseline pregnancy parameters were well balanced between the treatment groups. Second, we have a high rate of retention. Third, we use a reproductive and rapid molecular tool leading to the constitution of a homogenous cohort of pregnant women diagnosed with BV. Fourth, and in contrast to previous studies, we included women in early pregnancy, treated them quickly by adopting a point-of-care qPCR strategy, and ensured that the treatment was effective and that there was no recurrence, which is known to be high. With the advance of a molecular tools approach, our study has diagnosed and treated actually present molecular BV, whereas other methods would have included false-positives and unnecessary treatments.^6^ Finally, self-vaginal swabs are well accepted by women and give excellent results compared with speculum swabs, with a high resistance stability over time.^5,32,33^

The effectiveness of treatment of BV during pregnancy remains uncertain in our study, with less than 50% initial success and a recurrence rate of 32.6% (30 of 92). However, most patients included in previous studies without vaginal control swabs went untreated. By including vaginal swabs as a control in our study, we ensured greater efficacy of treatment. Classical antibiotic treatment, including metronidazole, is not always effective, with a high recurrence rate.^34,35^ In our study, the first-line treatment proposed was azithromycin despite this treatment having been rarely proposed or studied for BV. At the time of the construction of the study, azithromycin was shown to exhibit high in-vitro activity against microorganisms associated with BV.^36,37^ Additionally, azithromycin has greater in-vitro activity than metronidazole against *A vaginae*, of which strains resistant to metronidazole have been reported.^38^ Azithromycin is an inexpensive, well-tolerated antibiotic and is already administered in pregnancy for several conditions, such as sexually transmitted infections and intermittent preventive treatment for malaria.^39^

### Limitations

This study has limitations. The AuTop trial was initially designed as a cost-effectiveness analysis, with the primary end point being the incremental cost-effectiveness ratio. However, because of a nonsignificant clinical outcome (denominator of the ratio), the incremental cost-effectiveness ratio could not ultimately be calculated. Thus, effectiveness and cost outcomes were reported separately.

It was not possible to build a blinded study because women with positive test results in the experimental screen and treat group were required to receive treatment and iterative vaginal control swabs if positive from the study team. In addition, our scientific committee felt that it would be unethical to offer screening to all patients in both groups but not to treat patients in the control group.

In our study, loss to follow-up was less than 5%, but 44 of 242 patients screened with BV (18%) did not receive treatment. This reflects the difficulties of a randomized trial nested within routine practice in the care of pregnant women and may lower the effectiveness of the screen and treat strategy.

The composition of bacteria present in BV varies among individuals; other species may be frequently found. The possibility that some of these infections may have been treated by the antibiotics used was not investigated in our study. Although randomization should ensure that the groups are similar in terms of microbiota, this may be a limitation. In the study population, the burden of BV was lower than expected (7% instead of 10%), which may have resulted in an inconclusive statistical result for the primary intention-to-treat end point.^40,41,42^

The generalization of molecular diagnosis in clinical microbiology during the COVID-19 pandemic^43^ could encourage laboratories to carry out the diagnosis of BV by molecular biology based on real-time qPCR. This would make it possible to have a rational and reproducible diagnosis by overcoming the pitfalls of the Nugent score and the Amsel criteria.

## Conclusion

In this clinical trial of pregnant women at low risk of preterm birth, molecular screening and treatment for BV based on *A vaginae* (*Fv aginae*) and/or *G vaginalis* quantification did not significantly reduce preterm birth rates. Post hoc analysis suggests a benefit of screen and treat in low-risk nulliparous women, warranting further evaluation in this group.

## Notes

Supplement 1. Trial Protocol

Supplement 2. Statistical Analysis Plan

Supplement 3. **eFigure.** Schema of Treatment Algorithm **eTable 1.** Table of Primers and Probes Used in the Study **eMethods 1.** Molecular Analysis Procedure **eMethods 2.** European Patent **eTable 2.** Details of Cost Outcomes According to Randomly Assigned Groups in the AuTop Study **eTable 3.** Sensitivity Analyses With Complete Cases and With Missing Values Imputed Using the Worst-Case Scenario **eTable 4.** Characteristics of the Patients in the Subgroup of Nulliparous and Multiparous Women According to Randomly Assigned Group

Supplement 4. Group Information. Groupe de Recherche en Obstetrique et Gynécologie (GROG) Investigators

Supplement 5. Data Sharing Statement
