# Supplementary material for: Effectiveness and Costs of Molecular Screening and Treatment for Bacterial Vaginosis to Prevent Preterm Birth: The AuTop Randomized Clinical Trial
Source: JAMA Pediatr. 2023 Jul 17;177(9):894–902. doi: 10.1001/jamapediatrics.2023.2250 (PMC10352927; doi:10.1001/jamapediatrics.2023.2250)
Supplement: Supplement 1. — Trial Protocol [file jamapediatr-e232250-s001.pdf]

Medico-Economic Impact of Screening *Atopobium Vaginae* and *Gardnerella Vaginalis* by Molecular Biology using "Point-of-Care" testing During Pregnancy

Principal Investigator

Prof. Florence Bretelle

Assistance Publique - Hôpitaux de Marseille (AP-HM)

Gynépole Marseille

Table of Contents

I. Introduction 5
A. Preterm birth and the link between infection and preterm birth 5
B. Bacterial vaginosis (BV) 5
C. Risks associated with BV 6
D. Techniques for diagnosing vaginal flora anomalies 6
1. Standard technique(s) 7
2. Innovative technique 7
E. Treatment of BV during pregnancy and its recurrences 8
F. Rationale of the study 11
II. Objectives of the research 13
A. Main objective 13
B. Secondary objectives 14
III. Methodology 15
A. Choice of experimental design and rationale 15
B. Teams involved 15
C. Study population 16
1. Subject recruitment 16
2. Selection criteria 17
D. Study groups 18
1. Group A: screening using molecular biology techniques and treatment if positive 18
2. Group B: control group 19
E. Endpoints 19
1. Primary endpoint 19
2. Secondary endpoints 19
3. Medico-economic evaluation 21
4. Budget impact analysis 23
F. Expected number of people and justification 24
G. Participation period 25
IV. Research design and conduct 26
A. Division of labor 26
1. Research Steering Committee 26

2. Scientific Committee ... 26

3. Gynecology-Obstetrics departments of participating centers ... 26

4. Role of the midwives ... 27

5. Methodological Support Unit for Clinical Research and Medico-Economic Evaluation ... 27

6. The Clinical Research and Innovation Department of the AP-HM ... 27

B. Follow-up organization ... 27

1. Inclusion consultation ... 27

2. Bacteriological analysis ... 28

3. Therapeutic management and vaginal self-sampling ... 29

4. Management of treatment failure and recurrence of vaginal flora anomalies ... 29

5. Study exit ... 30

V. Data processing ... 30

A. Data collection ... 30

1. Organization ... 30

2. Data collected at inclusion ... 30

3. Data collected at the time of delivery ... 31

4. Data collected when exiting study ... 31

5. Data collected before the study was set up and at the end of the study ... 32

B. Quality control ... 32

C. Data entry ... 32

D. Statistical analysis of the data ... 32

1. Test populations ... 32

2. Population description, initial comparability of the groups ... 32

3. Analysis of the primary endpoint: incremental cost-effectiveness ratio and addressing uncertainty ... 33

4. Analysis of the secondary endpoints ... 33

5. Multivariate analyses ... 34

E. Management of serious adverse events (SAEs) ... 34

VIII. Total expected duration of the research ... 34

IX. Expected outcomes – Prospects ... 35

X. Clinical trial vigilance ... 35

A. Definitions ... 35

1. Adverse event (AE) ... **Erreur ! Signet non défini.**

2. Serious adverse event (SAE) ... 35

3. Adverse reaction to an experimental drug (AE) 36
4. Unexpected adverse reaction 36
B. Investigator's responsibilities 36
1. Procedures for detecting and documenting adverse events 36
2. Reporting of serious adverse events 36
3. Assessment of causality 37
4. Reporting period 37
C. Sponsor’s responsibilities 37
1. Reporting of unexpected serious adverse reactions 37
2. Safety developments reporting 38
3. Annual safety report 38
4. Independent monitoring committee 38
XI. Publication rules 38
XII. Legal and ethical aspects 38
XIII. References 40

I. Introduction

A. Preterm birth and the link between infection and preterm birth

Preterm birth is defined as a delivery before 37 weeks of gestation. It is the main cause of neonatal mortality and morbidity. The risk of mortality and morbidity is inversely related to the gestational age at birth.

In France, the preterm birth rate remains stable despite enhanced prevention measures (notably, the 2005-2007 perinatal plan) and the development of tocolytic treatments. The preterm birth rate is currently estimated at 7.4% amongst the general population and 6.3% for singleton pregnancies (2010 perinatal survey). Spontaneous preterm births[1] (Menard 2012) account for 65 to 85% of preterm births.

Preterm birth is linked to multiple factors. In addition to vaginal infection and bacterial vaginosis (BV), there are many risk factors: age, body mass index, ethnicity, history of preterm delivery, multiple pregnancies, pregnancies induced by medically-assisted procreation, smoking, hypertension, fetal growth restriction (FGR), etc.

Among these risk factors, vaginal infection is the main cause of spontaneous preterm birth (delivery before 37 weeks of gestation), accounting for 40 to 70% of cases according to publications (Goldenberg 2000). **The majority of these patients have no prior medical conditions** and no risk factors that would help predict preterm birth (90%).

The link between preterm delivery and BV is currently acknowledged. **This link is even stronger the earlier the BV diagnosis is made** during pregnancy (Andrews 2000; Goldenberg 2000, 2006).

B. Bacterial vaginosis (BV)

The vaginal cavity is naturally colonized by bacteria: lactobacilli *(Lactobacillus crispatus, L. jensenii, L. iners, L. gasseri*). They ensure the vaginal ecosystem remains normal by producing hydrogen peroxide, lactic acid and bacterial-growth inhibitors (bactericides), but also by adhering to the vaginal walls (biofilm) and by maintaining the vaginal pH between 3.8 and 4.5. All of these mechanisms play a part in inhibiting multiplication of bacteria and maintaining bacteria that are found in the normal state *(Corynebacterium* spp., *Streptococcus* spp., *Enterococcus* spp., *G. vaginalis, A. vaginae, Mobiluncus* spp., *Candida albicans, etc.).*

Disruption of the vaginal ecosystem is characterized by the replacement of the lactobacilli of the normal flora by polymicrobial flora including *G. vaginalis*, (Thorsen 1998) *A. vaginae, Mycoplasma hominis, Mobiluncus* spp., and other anaerobic bacteria. Intermediate flora and BV are detected depending on the severity of the imbalance of the vaginal flora (Donders 2007).

While vaginal discharge and odor are the most common symptoms associated with BV diagnosis, about half of women with BV are asymptomatic (Klebanoff 2004).

The prevalence of vaginal flora imbalance in the population varies from 5 to 55% (Cauci 2002, Marrazzo 2002, Hay 1994, Cristiano 1996). Geographical origin, ethnicity and socio-

---

[1] Spontaneous preterm births are characterized by patients going into labor before 37 weeks of gestation, as opposed to preterm births resulting mainly from Caesarean section due to maternal or fetal factors.

economic conditions affect the prevalence of BV in pregnancy (Thorsen 2006, Larsson 2007, Trabert 2007). In the USA, a case-control study found rates of BV of around 9% in Caucasian women compared to 23% in black women, 16% in Hispanic women and 6% in Asian patients (Hay 1994). Similarly, this prevalence depends on the trimester of the pregnancy: for example, a screening survey of London patients showed a prevalence of 12% in those with a pregnancy of less than 28 weeks of gestation (Hay 1994) compared with 20% at 30 weeks. Lastly, unlike the high prevalence (18% to 55%) in North American populations, in Europe and France, the prevalence is lower (5% to 14%). Less than 10% of patients with normal flora at the beginning of pregnancy will develop BV (Vogel 2006).

C. Risks associated with BV

During pregnancy, BV is associated with increased risk of obstetric complications: premature rupture of the membranes (PROM), preterm delivery, chorioamnionitis, low-birth-weight (LBW) infants (Svare 2006).

The association between BV and preterm births has been widely published over the last 20 years. From the meta-analyses, it should be noted that pregnant women with BV are twice at-risk for preterm birth than women without BV (OR: 2.16, 95% CI: 1.56-3.00) (Leitich 2007). This risk is higher the earlier the diagnosis is made, especially before 16 weeks' gestation (Leitich 2003).

For pregnancies complicated by a risk of preterm delivery, the presence of BV is detrimental as it doubles the risk of delivery before 37 weeks' gestation (OR: 2.38, 95% CI: 1.02-5.58) (Leitich 2007).

In addition to preterm delivery, data from meta-analyses shows that the presence of BV increases the risk of late miscarriages (OR: 6.32, 95% CI: 3.65-10.94) and maternal infection in the postpartum period (OR: 2.53, 95% CI: 1.26-5.08). This risk is higher in terms of very preterm birth and miscarriages (OR 5.3, 95% CI: 2.1-12.9 and OR 6.6, 95% CI: 2.1-20.9 respectively) (Donders 2009). This risk varies by ethnicity. Black patients are three times more likely to have BV, which would explain the 50% increase in the risk of preterm delivery in this group.

While BV is associated with prematurity, no association has been demonstrated between BV, neonatal infection and perinatal mortality.

A change in the vaginal flora during pregnancy is also a factor in preterm delivery (Carey 2005). A recent study in a high-risk population identified a rate of BV of about 7% and 21% of intermediate flora (Goffinet 2005).

Our team has recently shown that the presence of high levels of *A. vaginae* is a risk factor for preterm delivery and shortens the time to delivery (Leitich 2007, Menard 2010 B).

D. Diagnostic techniques for vaginal flora anomalies

The diagnosis of BV has always been problematic and has led to tests of questionable sensitivity and specificity (Lamont 2005).

Initially, gynaecologists used Amsel's diagnostic criteria. This diagnostic criterion is not practiced in France. The Nugent score is the standard method in France (Appendix 3).

1. Standard technique(s)

Vaginal flora abnormalities are classically determined using Amsel's clinical criteria or Nugent's score in a patient with symptoms in the vulvar and vaginal areas.

a. Clinical definition

Bacterial vaginosis (BV) is clinically defined by the presence of 3 of the 4 Amsel criteria (Amsel 1983).

- homogeneous milky vaginal discharge adhering to the vaginal walls
- vaginal pH >4.5
- foul vaginal odor
- clue cells on direct microscopic examination

Of the 4 criteria, the first 3 will be noted by the doctor during the consultation. The appearance of the discharge is observed upon examination using a speculum. The determination of the vaginal pH is carried out using a pH paper that is placed in contact with vaginal secretions. A foul vaginal odor is noted during the examination or detected by a test using potassium hydroxide, which involves preparing a slide of vaginal discharge and adding a drop of 10% potassium hydroxide (whiff test). The combined presence of the first three criteria (milky discharge adhering to the vaginal walls; vaginal pH >4.5; foul vaginal odor) defines an imbalance in the vaginal flora.

b. Microbiological definition

The paraclinical test used to define vaginal flora anomalies is Gram stain and reading the slide under a microscope using 1000x objective, followed by interpretation according to criteria defined by the Nugent score (Nugent 1991, Appendix 3).

The Nugent score is established by Gram stain by characterizing the presence or absence of 3 types of microorganisms in the vaginal flora: *Lactobacillus* spp., *G. vaginalis* and *Bacteroides* spp., *Mobiluncus* spp.. The total Nugent score is the sum of the 3 sub-scores assigned according to the level of absence or presence of each microorganism (see Appendix 3). The Nugent score results in 3 categories: normal, intermediate or abnormal vaginal flora.

The Nugent test is very problematic because the Gram stained slide must be read by an experienced operator, and performed on a fresh swab. Experience shows that, very often, the time it takes to transport the swabs causes them to dry out, making it difficult to perform the Nugent test, and that there is a difference in interpretation among operators examining the same slide stained in the same way. For 21st century diagnostic tests, these limits are not reasonably acceptable.

2. Innovative technique

This observation initially led the Gynecology-Obstetrics department and the microbiology laboratory of the AP-HM to work together to produce a modern reproducible, molecular test. The assessment of all microorganisms that were likely to be associated with vaginosis was carried out through an original endeavor that was published in the leading journal of infectious diseases (Menard 2010 A) (ranked class A in SIGAPS, Impact Factor=9.154), along with the filing for a patent (European Patent Office No. 2087134).

Unquestionably, the quantification of the number of *A. vaginae* copies is the element that has the most significant predictive value and is sufficient to create a cut-off point that specifically differentiates between patients with vaginosis from those without vaginosis. The superiority of this single comparative test is considerable. In addition, the test includes an assessment of the quality of the sample by determining the concentration of the human albumin gene (gene of human origin).

This test has since been evaluated in several studies (Menard 2008, Menard 2010 A, Menard 2010 B, Menard 2012 A), which have shown its feasibility and reproducibility, including in self-collected samples, and on a broad series of patients.

We have also shown that 57% of flora classified as intermediate according to the Nugent score was in fact real BV (Ménard et al. 2008). Thus, molecular testing identifies a homogeneous population of patients with BV.

At international level, Fredricks' team was the first to characterize vaginal flora in molecular analysis (Fredricks 2005, Fredricks 2010).

Very recently, other teams have explored diagnosing BV through molecular analysis (Cartwright 2012). The authors also discovered excellent sensitivity and specificity. In the paper, where a combination of 3 microorganisms was being tested, it appears that *A. vaginae* is almost always present in BV. This has been verified in our work but also by other authors (Marconi 2012). We have indeed shown (Appendix 1) that in the event of BV, other microorganisms were present but in association with the presence of *A. vaginae* or *G. vaginalis*, hence the choice not to look for other microorganisms such as *Mycoplasma hominis*, *Ureaplasma urealyticum* or *Candida albicans*.

E. Treatment of BV during pregnancy and its recurrences

Currently in France, it is recommended that vaginosis and vaginal infections be screened and treated in the second trimester of pregnancy only if there is a history of preterm birth or late miscarriages (ANAES 2001). The contradictory results of the latest scientific articles on the subject do not make it possible, to date, to modify the current recommendations for screening and treating vaginal flora anomalies (ANAES 2001 and 2005).

The 2013 Cochrane Data Base update (21 studies and 7847 patients) does not recommend screening for BV in the absence of impact on preterm birth in either the low or high risk population (Brocklehurst 2013). However, this meta-analysis states that 2 studies, including intermediate flora and BV (Nugent score>4), show a reduction in preterm delivery before 37 gestational weeks (RR 0.53 CI [0.34-0.84] and miscarriages (RR 0.2 CI 0.04-0.89] (Lamont 2003 and Ugwumadu 2003).

	Population	Definition of vaginosis	Term inclusion	Treatment	Preterm birth impact
**Carey 2000 (n=1953)**	Mixed (12%)	Clinical & Nugent >7	8-22 WG control VS	Oral metronidazole	NS 1(0.8-1.2)
**Hauth 1995 (n=624)**	High-risk	Clinical and Gram	22-24 WG control VS	Metronidazole oral & erythromycin	31% vs. 49% **
**Morales 1994 (n=80)**	High-risk	Clinical	13-20 WG no control VS	Metronidazole Placebo 100 mg vitamin C oral	39% vs. 18% **
**Odendaal 2002**	Mixed	Clinical and Gram	15-26 WG	Metronidazole Placebo 100 mg vitamin C oral	Preterm birth increase metronidazole (43% vs. 24%)
**Ugwumadu 2003 (n=6129)**	Mixed (15.7%)	Nugent > 4	22 WG no control VS	Clindamycin oral	5.3 vs. 15.7% **
**McDonald 1997 (n=879)**	Mixed (7.5%)	Gram and culture	16-26 control VS at 29 WG	Metronidazole	NS (7.2 vs. 7.5%) risk sub-group 9.1 vs 41.7%
**Lamont 2003 (n=409)**	Mixed (10%)	Nugent > 4	13-20 WG control VS (20-24 WG)	Clindamycin vaginal	10% vs. 4% **
**Kiss 2004 (n=4229)**	Low-risk	Nugent >7	15-20 WG	Clindamycin vaginal (oral if failure)	3.0% *vs.* 5.3%, ***

Table 1: A non-exhaustive review of the main publications on the impact of treating BV during pregnancy. Vaginal Swab: VS; WG: weeks' gestation.

In high-risk populations, the literature review shows no efficacy of treatment for BV (Okun 2005, Lamont 2005, Brocklehurst 2013).

The studies in which treatment was effective were those of Hauth et al. and Morales et al. based on very high risk of preterm birth. The numbers in the Morales et al. study are small and the definition of vaginosis is mainly based on clinical criteria (the asymptomatic nature of the patient could be questioned) (Morales 1994).

In the low-risk and intermediate-risk population, the results are as follows. In Ugawumadu's study (Ugawumadu 2003), intermediate flora was treated. The impact of the treatment on the intermediate flora was greater the higher the Nugent score. In 2004, another study published a few months later in the *British Medical Journal* demonstrated, for the first time, the effectiveness of screening combined with treatment of BV and asymptomatic vaginal infections during pregnancy, in terms of impact on preterm birth and on the decrease in the proportion of low-birth-weight babies (screened-treated group versus unscreened-untreated group) (Kiss 2004).

The **meta-analyses** (McDonald 2007, Okun 2005, Lamont 2005, Brocklehurst 2013) **differ in their results** depending on the studies selected. In summary, the discrepancies between these meta-analyses highlight their heterogeneity, the difficulty of reaching a definitive conclusion on the subject and **the need for further studies**.

Why are the results of therapeutic trials on flora anomalies divergent?

Explanations for the lack of a positive impact on preterm birth vary depending on the studies:

1/ **Differences in the choice of treatments** are a major factor in the difficulty of the studies' conclusions. The type of antibiotic therapy and the methods of administration (oral, vaginal) vary. Moreover, few studies evaluate the effectiveness of the treatments (Lamont 2003, Mac Donald 1997).

However, there are international and national recommendations for the use of metronidazole or clindamycin during and outside pregnancy (WHO 2005, US Centers for Disease Control and Prevention 2006). In France, the available and recommended treatment for pregnancies at high risk of preterm delivery when BV is detected is oral metronidazole (ANAES 2001).

The clinical and microbiological arguments for the choice of treatment are detailed in Appendix 4. In conclusion, clinical studies do not allow us to choose one treatment over another. As far as microbiological aspects are concerned, there are strong arguments for seeking treatments other than those currently in use (resistance to the usual treatments). Azithromycin has been shown to be effective against *A. vaginae* and *G. vaginalis* and has a half-life that allows for a shorter treatment period (De Backer 2006). The treatment has the advantage of being effective against mycoplasma. The choice of azithromycin seemed logical based on the literature review (Appendix 4).
It should be noted, however, that azithromycin is being evaluated off-label for the treatment of BV.

2/ **Another possible explanation hinges on the notion of recurrence**. In the event of BV, *A. vaginae* and *G. vaginalis* produce a pathological biofilm that is destroyed at the time of treatment and is reconstituted after any treatment is stopped. As a result, the risk of recurrence is high, ranging from 28 to 50% depending on the study. The further away from the initial treatment, the higher the rate. Thus, the recurrence rate is 35% at 1 month, 50% at 3 months and up to 70% at 12 months after treatment (Bradshaw 2006). Recurrences expose patients to the complications mentioned above and lead to further consultations and treatment. The risk of recurrence justifies follow-up after treatment, particularly during pregnancy (ANAES 2001). Post-treatment follow-up adds to the cost of treating this condition.

Therefore, the failure of some treatment protocols could be explained by the **fact that screening for BV recurrence is not taken into account or is taken into account at a late stage**.

3/ **The term of the pregnancy** at the time of treatment is also a major factor. If treatment is provided at a very late stage, the ascending infection causes infection of the membranes which can lead to premature delivery. This weakened status can lead to premature rupture of the membranes. An imbalance in the flora can similarly foster an ascending infection. Screening at the end of the first trimester would be occurring downstream of early spontaneous fetal discharge and before the pathogenic effects of a vaginal infection or BV on the pregnancy has been established.

4/ Lastly, the Gram stain-based Nugent score **identification technique** is subject to inter- and intra-operator variability (Menard 2008). Standardization and quantification of BV by PCR makes it possible to characterize the vaginal flora. This identification technique has been published in the *New England Journal of Medicine*, which emphasizes the low relevance of the Nugent score. (Fredricks 2005).

Moreover, molecular analysis makes it possible to diagnose BV in intermediate flora (Nugent

score between 4-7): we have shown that 57% of flora classified as intermediate is in fact fide BV (Menard et al. 2008). Therefore, **previous studies based on NUGENT score >7 potentially failed to identify 57% of patients who should have been included, and those including a score >4 erroneously included 43% of patients without BV** (Menard et al. 2008; Menard et al. 2010). This highlights the difficulty of drawing conclusions when treatment was not given to a proportion of patients who should have been treated.

F. Rationale for the study

A review of the literature and practices shows that conventional screening tests are heterogeneous, subjective and that treatment is insufficient if not combined with screening for recurrence. The literature as a whole, including meta-analyses, calls for further studies on homogeneous populations[2]. It seems to indicate **the value of systematic screening and treatment** of vaginal infections in pregnant women at the beginning of the second trimester of pregnancy. Systematic therapeutic management of these mostly asymptomatic infections should have **an impact in terms of preterm birth**. Today, it is important to be able to demonstrate **the benefit of this measure** and **the benefit of accounting for recurrences**.

Our work has already validated the comparison with the Amsel criteria and Nugent score. The diagnosis of BV will be carried out through **molecular analysis** using our patented tool, which has proven its feasibility and reproducibility (Mangot-Bertrand 2012, Menard 2008, Menard 2010 A Menard 2010 B, Menard 2012). A proportion of flora classified as "intermediate" (57%) will thus be **reclassified as BV**.

Rationale for Point-Of-Care or "POC":

In the current work, the aim is to incorporate this technique into POC (Point-Of-Care) laboratories. The development of a "Point of Care" rapid diagnostic molecular tool allows the clinician to have a **rapid result at the time of care** (Cohen-Bacrie 2011, Nougairede 2010).

Thus, all the various steps that allow the development of a test likely to serve as the rationale behind developing a test of this nature will emerge during the current study, which should be a decisive element in the search for an industrial partner. If demand for this type of examination were to materialize, partnering with the Méditerranée Infection Foundation in view of developing this breakthrough diagnostic tool and passing it on would become a priority.

The rapid delivery of results means that fewer patients are lost to follow-up and patients can be treated without delay. Gestational age at diagnosis of vaginosis is indeed a risk factor for preterm delivery or miscarriage.

Rationale for the self-sampling technique

Vaginal self-sampling is a simple and validated method of sampling. It has been successfully used for the molecular biology technique and the quantification of microorganisms involved in vaginal flora imbalance (Baay 2009). This sampling method is used, due to its acceptability and sensitivity, for screening sexually transmitted infections (Knox 2002, Ogilvie 2005, Petignat 2005, Anhang 2005, Chernesky 2005, Waller 2006). The vaginal self-sampling

---

[2] Antibiotics for treating bacterial vaginosis in pregnancy. McDonald HM, Brocklehurst P, Gordon A. **Published Online: January 19, 2011"** Antibiotics during pregnancy for overgrowth of abnormal bacteria in the birth canal does not reduce the risk of babies being born too early. Bacteria are normally present in the birth canal and are useful in maintaining the health of the vagina. However, if the numbers of abnormal bacteria increase, this may cause an unpleasant discharge and may cause some babies to be born too early. The review of 15 trials, involving 5888 women, found that antibiotics given to pregnant women reduced this overgrowth of bacteria, but did not reduce the numbers of babies who were born too early. The effect of earlier treatment needs to be studied in further trials."

method is compatible with pregnancy (Bresson 2006, Nelson 2003, Strauss 2005). A previous study carried out in our department showed an excellent correlation for real-time PCR quantification of *A. vaginae*, *G. vaginalis* and *Lactobacillus* spp. between the results obtained from self-collected vaginal swabs and physician-collected swabs (Menard 2010 A, B; Menard 2012 A and Tamalet 2010).

Risks related to the technique, expected complications

The risks associated with screening for BV are low. The self-sampling technique is acceptable, easily performed by patients and without risk of complications.

The risks are related to false positives (false positive rate of about 5% according to our previous studies) and are essentially those of macrolide treatment. This treatment is well tolerated with no expected side effects in either mother or child (Contraindications: allergy to macrolides, in combination with drugs containing cisapride, ergotamine or dihydroergotamine; possible adverse reactions to the drug: allergic skin reaction, nausea, vomiting, diarrhea, abdominal pain, candidiasis, dizziness, nervousness, convulsions (rare), increased transaminases, hepatitis (exceptional), hearing impairment; no teratogenic effect expected in pregnancy).

Given the 100% sensitivity of our tool, the expected false negative rate is zero. Patients with a "false negative" test will not be treated and will be managed in the usual way and therefore monitored as if they had not been screened.

Rationale for the implementation of a medico-economic assessment

In 2012, 822,000 births were recorded in France, according to the 2010 Perinatal Survey, 11.4% of patients had an obstetric history of prematurity or perinatal deaths. Thus, approximately 700,000 pregnant women presenting a low risk of preterm birth could benefit from screening for BV in France each year (Blondel 2012). Analysis through molecular biology that is rapid, reproducible and has good diagnostic performance could make it possible to widely deploy this screening method. Its implementation by Point-Of-Care (POC) raises the question of the existence or establishment of delocalized laboratories, which require investing in dedicated equipment and staff. Since the goal is to extend screening to a majority of, or even all, low-risk pregnant women, this raises the issue of the deployment of such laboratories throughout the country and their profitability in terms of service rendered. Currently, the activities of POC platforms are expected to develop around multiple molecular tests for which recourse to the POC is the relevant solution (rapid results obtained while undergoing care and allowing the clinician to optimize care by immediately initiating treatment). The equipment and/or staff could therefore be amortized through intensive use, thus ensuring economies of scale.

Today, microorganisms such as the influenza virus, enterovirus, streptococcus B, HSV and VZV viruses, pneumococcus, meningococcus and *Mycoplasma pneumoniae* are already analyzed in POC in Marseille in the two public academic teaching hospitals of Timone and Nord. A 2012 implementation of a POC laboratory in a remote area in Senegal, overseen by the IFR 48, CNRS-IRD UMR 7278 laboratory, demonstrated the feasibility of this type of deployment (Mediannikov 2012).

The POC concept was created about 20 years ago (Clerc 2010). The development of POC analyses and laboratories, particularly in the field of infectious diseases, has accelerated in recent years across the globe. This type of laboratory exists in the United States and Europe (Clerc 2012, Jenny 2010). An Australian team has even succeeded in developing a POC laboratory that can be deployed in the field (Inglis 2011). As far as the dissemination of this

technique is concerned, it should be noted that if the principle of the POC laboratory is not generalized, it is often due to a lack of staffing.

However, the molecular biology techniques used at POCs are currently performed in a large number of laboratories, which makes this technique relatively easy to disseminate.

In the particular case of the dissemination of screening for significant vaginal flora anomalies, reagents are subject to costs, thus adding to the cost of investing in staff and equipment required to operate the POC. To this effect, this systematic screening of low-risk pregnant women entails additional costs for the community compared to current practices, which are based on targeted screening using traditional methods, and which in reality are very rarely performed. In addition to the extra cost of systematic screening, there is the extra cost of diagnosing recurrences for patients who have been identified as positive and the extra cost of treating BV and its recurrences.

The mortality and morbidity associated with preterm birth place a significant economic burden on the finite resources of the community. Children born prematurely are at increased risk of neonatal adverse events and on average require more intensive and longer stays in neonatal units. Following this initial period of hospitalization, preterm children are more frequently hospitalized and have more frequent contact with health and social welfare professionals than children born at full term. Finally, in the long term, these children are at greater risk of disabilities, learning difficulties or behavioral problems that require appropriate and often significant care. A relatively large number of articles have been published on the economic consequences of preterm birth with different timelines ranging from the initial hospitalization period to the entire childhood period.

A recent literature review focusing on preterm deliveries between 32 and 36 weeks' gestation (Petrou 2012) has shown that for a child born preterm alive at birth, the average cost of care during the initial period of hospitalization could be between $5,041 and $35,635 depending on the study and the term, compared to a cost that could be between $1,334 and $3,860 for a child born at full term. The cost is higher the lower the term at the time of birth, up to $231,852. A study that estimated the costs incurred in the public sector up to the child's 18th birthday found that the incremental cost per survivor ranged from £7,612 for a birth at 36 weeks' gestation to £234,497 for a birth at 23 weeks' gestation (Mangham 2009; Kiss 2006).

Therefore, if our study shows that screening for BV and its treatment reduces the rate of preterm delivery from 4.3% to 3% in a low-risk population, substantial cost savings should be made and should undoubtedly fully justify the initial investment required for such screening: the medico-economic aspect of our study aims to evaluate to what extent and under what conditions.

II. Objectives of the research

A. Main objective

The main objective of the study is to evaluate the medico-economic impact, through a cost-effectiveness study, of a new screen-and-treat strategy for vaginal flora abnormalities before 20 weeks' gestation, in a population of pregnant women at low risk of preterm birth.

The screening strategy is based on screening for significant vaginal flora anomalies using molecular analysis (*A. vaginae* and *G. vaginalis* PCR by Point-Of-Care) after a self-collected vaginal swab. BV treatment will be offered for any positive result. The screen-and-treat strategy will be compared to a standard strategy of usual care.

B. Secondary objectives

The secondary objectives will be as follows:

- To assess the benefit of the screen-and-treat strategy for vaginal flora anomalies based on the following efficacy parameters: delivery before 26, 28 and 32 weeks' gestation and 37 weeks' gestation, ruptured membranes, fetal growth restriction, endometritis, corrected spontaneous preterm birth, risks of preterm birth;
- Compare the 2 groups in terms of the:
    - duration of the mother's hospital stay before and after the birth;
    - duration the child's hospital stay in the 6 months following the birth, including conventional hospitalization, hospitalization in neonatology and hospitalization at home;
    - neonatal mortality and morbidity in the first 6 months of life,
    - spontaneous abortion rate before 14 weeks' gestation (13 and 6 days) and before 22 weeks' gestation;
    - quality of the mother's life of at 6 months postpartum.
- To determine the frequency of BV recurrence and to document links with the demographic and clinical parameters of women (age, smoking, vaginal hygiene, sexual activity, contraception, etc.);
- To determine the effectiveness of the treatment on the flora and on the levels of *A. vaginae* and *G. vaginalis*;
- To evaluate the real average cost of Point-of-Care molecular analysis of a vaginal swab for BV;
- To assess the medico-economic impact (cost-effectiveness) for different terms of preterm delivery (26-28 weeks' gestation, 32 weeks' gestation, 37 weeks' gestation);
- To evaluate the cost to the community of caring for a premature birth over the first 6 months of the child's life;
- Conducting a budgetary impact analysis of the innovation;
- To analyze the subgroup of patients screened by traditional methods (Nugent score, etc.) for BV in Group B:
    - document the frequency/type of real-life screening for BV, screening for recurrence and treatment;
    - measure the determining factors (age, socio-professional category, clinical status, prescriber, etc.);
- To have process indicators in order to measure the feasibility of the dissemination of the innovative technique across the French territory and to carry out an analysis of the budgetary impact of the innovation for the payer.

III. Methodology

The methodology used in the study is in line with the recommendations of the Consolidated Standards of Reporting Trials Statement (CONSORT, http:// www.consort-statement.org/consort-statement/).

A. Choice of experimental design and rationale

This is **a prospective, randomized, open-label comparative study** comparing 2 groups of pregnancy management in a population of pregnant women at low risk of preterm birth.

The recruitment of subjects will be carried out on a prospective basis. The 2 strategies/groups are detailed in chapter D of the methodology section of this project.

Screen-and-Treat Innovative Strategy (Group A): patients **systematically screened for** BV before 20 weeks' gestation by means of a vaginal swab analyzed by the innovative technique, whose result will be disclosed. If positive, appropriate treatment will be prescribed.

Control Group or Standard Strategy (Group B): patients not systematically screened for BV/usual care group.

This set-up allows for optimal experimental design and level of evidence: randomized controlled trial.

B. Teams involved

The associated departments are: the Gynecology-Obstetrics department, the Clinical Investigation Center (Nord public academic teaching hospital) of the AP-HM, the PérinatSud network, the Gynecology-Obstetrics department of Poissy-st-Germain (Prof. Rozenberg), Angers public academic teaching hospital (Dr Sentilhles) the Armand Trousseau public academic teaching hospital (Prof. G Kayem), Robert Debré public academic teaching hospital (Dr Thomas Schmitz), Public academic teaching hospital of Clamart Hospital (Prof. Alexandra Benachi), Public academic teaching hospital of Kremlin Bicêtre (Prof. Marie Victoire Senat), Public academic hospital of Créteil (Prof. Haddad - Dr Menard) Maternal and Infant Protection Unit of the Conseil Général du Val de Marne (Dr JP Ménard), Public academic teaching hospital of Nice Hospital (Prof. Bongain), Public academic hospital of Nîmes (Prof. Mares), Public academic hospital of Aubagne (Dr Nawal Chenni), Public academic hospital of Aix-en-Provence (Dr Xavier Danoy), Bouchard Private Hospital in Marseille (Dr Nadia Slim) Saint-Joseph Private Hospital in Marseille (Dr Raoul Desbrière), Public academic hospital of Toulon (Dr Franck Mauviel), Public academic teaching hospital of Saint-Etienne (Prof. Céline Chauleur), Pau Hospital (Dr Caroline Bohec), Public academic teaching hospital of Guadeloupe (Dr Philippe Kadhel), Public academic teaching hospital of Martinique (Dr Jean-Luc Volumenie).

The teams involved are particularly committed to the fight against preterm birth.

Two Point-of-Care centers will perform the analyses using the patented reproducible tool: one center in the Paris area, **the Biochemistry and Molecular Biology Laboratory of the Paris-Ile-de-France-Ouest Faculty of Medicine, UPRES EA 2493, University of Versailles Saint Quentin en Yvelines, Poissy-St-Germain General Hospital (Dr Serazin)**, which will centralize the samples from the Ile de France region, while the South will be managed by the **Microbiology Federation of the AP-HM located at the Timone public**

academic teaching hospital (Dr F. Fenollar, Rickettsia Unit of Prof. D. Raoult, UMR7278, Faculty of Medicine, Marseille).

The economists associated with this study are:

- Ms. Cécile Fortanier, Research Engineer, Methodological Support Unit for Clinical Research and Economic Evaluation, Clinical Research and Innovation Department, AP-HM led by Prof. Pascal Auquier (EA3279, Evaluation of Public Health, Faculty of Medicine, Méditerranée University, 27 Boulevard Jean Moulin, Marseille cedex 05, 13385 France).
- Ms. Carole Siani, Senior Lecturer in Economics and Accredited Research Director, Claude Bernard University Lyon 1, ERIC Laboratory for Knowledge Storage, Representation and Engineering (EA3083) Institute of Pharmaceutical and Biological Sciences (ISPB)

The medical methodologist associated with this study is Dr Karine Baumstarck, Methodological Support Unit for Clinical Research and Economic Evaluation, Clinical Research and Innovation Department, AP-HM led by Prof. Pascal Auquier (EA3279, Evaluation of Public Health, Faculty of Medicine, Méditerranée University, 27 Boulevard Jean Moulin, Marseille cedex 05, 13385 France).

The statistician associated with this study is Mr. Anderson Loundou, Methodological Support Unit for Clinical Research and Economic Evaluation, Clinical Research and Innovation Department, AP-HM led by Prof. Pascal Auquier (EA3279, Evaluation of Public Health, Faculty of Medicine, Méditerranée University, 27 Boulevard Jean Moulin, Marseille cedex 05, 13385 France).

The coordinating clinical study technician is Mr. Jean-François Cocallemen.

The Clinical Research and Innovation Department of the AP-HM is represented for the monitoring of this study by Ms. Kahéna Amichi under the supervision of Mr. Loic Mondoloni.

The team of investigators and associated economists and methodologists sought an external expertise procedure for the latest version of this project implemented by Ms. Christel Castelli, Head of the Medico-Economics Unit of the BESPIM Department of the Nîmes Public academic teaching hospital, as part of the partnerships set up at GIRCI Sud Méditerranée Axe Innovation.

C. Study population

1. Subject recruitment

Pregnant women over 18 years of age who come to the study sites before 20 weeks' gestation will be invited to participate.

All doctors in the departments, who have been declared as investigating doctors, as well as midwives associated with the project, will be able to include subjects.

During the inclusion visit with the center's midwife and/or the investigating physician, after verification of the inclusion criteria and obtaining informed consent, the patient will be assigned to one of the 2 groups using the randomization list pre-established via CleanWeb.

- if the patient is assigned to **Group A (Screen-and-Treat Group):** in addition to the "molecular biology-based screening and treatment in case of testing positive," a

vaginal self-sampling kit will be provided during the same visit. The sample will be sent by the midwife to the POC laboratory. Data will be collected by the midwife through questioning the patient and consulting her medical record.

– if the patient is assigned to **Group B (Control Group):** “No screening/usual care.” During the same visit, data will be collected by the midwife through questioning the patient and consulting her medical record.

General practitioners, gynaecologists, midwives, OB-GYNs and laboratories performing screening for Down syndrome (trisomy 21) will be informed of this study. Posters and a website will be made available to patients. Health insurance companies will be contacted to offer this screening to patients.

Patients may be invited to participate especially when screening for Down syndrome, which has been in place in France in the first trimester (between 11 and 13 weeks’ gestation and 6 days) since January 2010, as well as at the time of declaration of pregnancy up to 20 weeks’ gestation. The midwives recruited for this project will open first-trimester consultations to allow them as well to include the 6800 patients.

2. Selection criteria

a. Inclusion criteria

- Pregnant women over 18 years of age before 20 weeks of gestation, regardless of gender or prior pregnancies;
- Woman who has understood the study process and objectives and agreed to sign an informed consent form;
- Without a history of preterm delivery or miscarriage (low-risk preterm birth population);
- Without significant high-risk factors for preterm birth: insulin-dependent diabetes, systemic lupus erythematosus, hypertension, uterine malformation, conisation, or multiple pregnancies;
- No pre-existing hypertension;
- Asymptomatic or symptomatic with regard to the diagnosis of BV.

b. Exclusion criteria

- Female minors (under 18);
- Woman of legal age under legal protection;
- Women deprived of their freedom for administrative or legal reasons;
- Woman who has not signed a consent form;
- At risk of preterm birth, at risk of miscarriage;
- Ectopic pregnancy;
- Non-evolutive pregnancy;

- For patients receiving azithromycin: History of allergic reaction to azithromycin, erythromycin, any other macrolide or any of the excipients, association with ergot alkaloids: dihydroergotamine, ergotamine, association with cisapride, association with colchicine, acute hepatic impairment;

- For patients receiving amoxicillin: allergy to beta-lactam antibiotics (penicillins, cephalosporins), allergy to one of the components of the drug, combination with methotrexate.

c. Exclusion criteria

- Woman withdrawing consent during the study.

D. Study groups

The randomization list will be established prior to the implementation of the study based on a 1:1 allocation ratio. It will be developed under the responsibility of the Public Health and Medical Information Department (Dr Karine Baumstarck, Methodological Support Unit for Clinical and Epidemiological Research, AP-HM). The selected method consists of patient blocks permuted per stratum (blocks of 6 subjects). The stratum chosen is represented by the center, in order to have the same proportion of each category in each center and thus minimize the center effect.

The procedure used for each inclusion is as follows: the investigating physician effects an inclusion via the CleanWeb interface. The group assignment for this inclusion is provided by CleanWeb.

1. Group A: screening using molecular biology techniques and treatment if positive

After randomization to Group A, the patient will perform the self-sampling during the inclusion visit. The dry swab will be sent to the POC center either by internal courier at the AP-HM and Poissy-st-Germain sites, or in a pre-stamped envelope to the nearest POC center. The dry swab shall be prepared for analysis using the technique described below (section V.B.2.b).

The results obtained using molecular biology techniques will be returned to the patient and the doctor. The manner in which the results are delivered will be adapted to each center.

A patient with a negative result at inclusion will not have another sample taken.

The positivity of the result will be defined based on molecular analysis only and in accordance with previous publications (Menard 2008). Evidence of *A. vaginae* > 10^5 copies/mL and/or *G. vaginalis* >10^5 copies/mL defines a positive test at the POC. The BV diagnosis is made when BV has been defined as *A. vaginae* $\geq 10^8$ copies/mL and/or a *G. vaginalis* load $\geq 10^9$ copies/mL and in this case treatment will be initiated. The patient will be treated with azithromycin 2 g orally (1 g at D1 and 1 g at D3 in one dose); in case of contraindication to azithromycin, a treatment with amoxicillin 2 g per day for 7 days will be

proposed.

Azithromycin and amoxicillin are being evaluated off-label for the treatment of BV.

Then, 3 control samples after the inclusion sample (D0) will be taken at D18, D48 and D78. Each patient who tests positive will therefore have a total of 4 samples taken. Treatment failure, cure or recurrence may in this case be characterized and will be managed based on the procedures defined in Chapter V. B. 4.

2. Group B: Control Group

No patients will be screened using molecular biology techniques in this group (not yet commercially available).

The management of these patients is a matter of routine for health professionals. They will be free to prescribe a standard vaginal swab if symptoms are present and to treat their patients according to their usual protocols. No routine sampling is recommended in the absence of a history.

The results of these samples and treatments will be recorded and included in the analysis of the results.

E. Endpoints

1. Primary endpoint

The primary endpoint will be the incremental cost-effectiveness ratio between the two groups corresponding to the cost per avoided preterm birth before 37 weeks.

The measurement of this criterion is detailed below in section IV.E.3. Medico-economic evaluation.

2. Secondary endpoints

The secondary endpoints will be:

- the rates of delivery before 26, 28, 32 and 37 weeks of gestation.
- the rate of rupture of the membranes defined as clean break with evident flow of amniotic fluid and/or a positive Amnicator, AmniSure, Actim PROM ((IGFBP-1) test.
- the rate of fetal growth restriction defined as an abdominal and/or femoral circumference measurement < 5th percentile for the gestational age (CNGOF curve, Créquat 2000).
- the rate of endometritis. Endometritis is characterized by a uterus that is painful on mobilization, the presence of purulent vaginal discharge (with the presence of altered leukocytes on vaginal swabbing) and hyperthermia >38°C requiring antibiotic therapy (with a negative result upon cytobacteriologic examination of the urine).
- The rate of corrected preterm birth (excluding preterm birth induced for exclusive maternal or fetal causes unrelated to the risk of preterm birth, rupture of membranes or chorioamniotitis) calculated as follows. Patients with caesarean section or labor induced before 37 weeks' gestation for any of the following reasons: preeclampsia, retroplacental hematoma, fetal heart rhythm abnormality, fetal growth restriction or death in utero of vascular origin, medical termination of pregnancy for fetal

malformation or chromosomal abnormalities will be excluded from this adjusted preterm birth rate.

- the rate of risk of preterm birth defined by uterine contractions occurring before 37 weeks' gestation and/or a cervical length of less than 25 mm on vaginal ultrasound.
- the total length, antepartum and postpartum, of hospitalization for mother and newborn in number of days (including conventional hospitalization, day hospitalization and hospitalization at home, hospitalization in neonatology or intensive care unit). All periods of hospitalization will be counted up to 6 months after the birth.
- neonatal morbidity will be assessed over the first 6 months of the child's life, by the occurrence of the following clinical events: respiratory distress syndrome, bronchopulmonary dysplasia, rate of `intraventricular hemorrhage, periventricular leukomalacia, necrotizing enterocolitis, sepsis, retinopathy of prematurity, newborns admitted to intensive care unit, mechanical ventilation, length of stay in intensive care unit, maternal side effects, frequency of fetal congenital anomalies.
- the rate of death after 22 weeks' gestation and within 6 months of delivery (neonatal mortality)[3].
- the spontaneous abortion rate before 14 weeks' gestation (13 and 6 days) and before 22 weeks' gestation.
- The quality of the mother's life at 6 months postpartum. The subjects' quality of life will be assessed using a generic questionnaire, the SF-12 version 2. It is the shortened version of a generic self-administered questionnaire, the SF-36 (Leplege, Mesbah et al. 1995; Leplege, Ecosse et al. 1998, Ware et al. 2002). The SF-36 has been widely disseminated and validated internationally in a variety of contexts. The short version has 12 items.
- the rate of recurrence of BV determined by molecular analysis during follow-up of positive patients and defined as a positive result after a negative result control in this group of patients.
- the effectiveness of the treatment assessed by comparing the rate of *A. vaginae*, *G. vaginalis* before and after treatment.
- average costs for each cost factor: cost of molecular biology techniques, cost of hospitalization, cost of BV treatment, cost of treatment of recurrences, cost of traditional screening methods, cost of management of preterm birth over the first 6 months, productivity losses as well as average total costs. The measures of these criteria are detailed below in section IV.E.3. Medico-economic evaluation.
- incremental cost-effectiveness ratios for preterm delivery terms at 32, 28 and 26 weeks' gestation. The measures of these criteria are detailed in section IV.E.3.
- the proportion and type of screening for BV and screening for BV recurrence performed by traditional methods (Nugent score, etc.) for the standard strategy (analysis of the subgroup of patients screened in Group B);

---

[3] Viability is defined by the WHO at 22 weeks' gestation; this duration varies depending on the publication. Our study uses 25 weeks' gestation to define fetal viability.

- the proportion and type of management of BV and its recurrence detected by traditional methods (Nugent score, etc.) for the standard strategy (analysis of the subgroup of patients screened in Group B);
- process indicators as regards dissemination of the technique across the country and budgetary impact. The measures of these criteria are detailed in section IV.E.4 below.

3. **Medico-economic evaluation**

The aim of the medico-economic evaluation is to inform choices in order to optimise the use of the limited resources available to society. The aim is to determine which of a range of possible strategies can be considered optimal not only in terms of medical effectiveness, but also in terms of the resources needed to implement it. It is based on a standardized methodology that requires specification of the point of view adopted for the analysis, the target population, the choice of comparator, the measurement of consequences, the categories of cost measured, the choice of unit costs, the follow-up period, the consideration of discounting procedures and accounting for uncertainty (CES 2003; Drummond 2005, HAS 2011).

a. Type of analysis

The medico-economic evaluation will be carried out from the point of view of the French healthcare system. This perspective is consistent with the objectives of the PRME (medico-economic research program of the French health ministry) invitation to tender. We will document the hospital resources mobilized for screening and treatment, as well as the outpatient and hospital-related consequences of the screen-and-treat strategy until delivery. In addition, we have chosen to also include in this analysis the indirect costs related to work stoppages, which can be a significant cost factor from a community standpoint.

The primary analysis in this project will be a cost-effectiveness analysis that will document the incremental cost-effectiveness ratio between the two groups in relation to the primary efficacy criterion identified as relevant by clinicians for the evaluation of this innovation. The choice of this criterion was discussed among the investigating physicians and with the economist. As this is a screen-and-treat strategy for pregnant women, the outcome criterion chosen was agreed upon by the investigating physicians: it is the rate of preterm delivery before 37 weeks' gestation (Beaino 2011, Theunissen 2001, Berbis 2012, Zwicker 2008).

The incremental cost-effectiveness ratio will be the cost per preterm delivery before 37 weeks' gestation avoided.

The time frame of the evaluation covers both the period of the intervention under study (i.e. pregnancy) and that of the occurrence of the event being measured (i.e. delivery, preterm or not). Therefore, the costs of implementing the two strategies under comparison will be measured over the period from the inclusion of pregnant patients (before 20 weeks' gestation) until the birth of the child (approximately 6 months of pregnancy). As the timeline is less than one year, the data will not require an updating procedure.

b. Cost factors for implementing the two strategies

The cost factors assessed will relate to the resources that are likely to vary between the two strategies being compared within the timeframe. These will be the following direct medical costs associated with the implementation of the two strategies**:**

- The cost of the initial systematic screening per POC PCR (Group A) including any additional procedures that may have been necessary to obtain a result (control procedures, etc.);
- The cost of screening for recurrence in patients who test positive after receiving a treatment (Group A) including any additional procedures that may have been necessary to obtain a result (control procedures, etc.);
- The cost of treating patients who test positive (Group A) and the cost of treating recurrences (Group A).
- The costs of any initial screening (Nugent score, etc.) and of any recurrences and treatments according to the usual practices of the professionals excluding molecular biology techniques (Group B);
- The costs of hospitalizations during pregnancies and of consultations.

Each of these costs will be measured in precise quantities consumed per patient: number of lab tests per POC PCR required for initial systematic screening and possible recurrence screening; number of tests per standard techniques (additional consultation); duration and type of treatments; number and duration of hospital stays. These physical quantities will be recorded in the e-CRF.

They will then be costed by assigning them a unit cost.

c. Unit cost of POC PCR screening

A detailed observation of the resources consumed during a representative number of procedures (in Marseille and Poissy) will be carried out: consumables, equipment operation duration, intervention duration for the various categories of staff, room occupancy duration. Consumables and staff interventions will be subject to costing based on negotiated prices and average salaries at the institutions.

For the costing of the cost linked to operating equipment (including maintenance), we will rely on the methodological considerations developed in the literature (Lucey 2002, Edejer 2003): an economic approach to the evaluation of capital costs combining depreciation cost and opportunity cost will be preferred. An “equivalent annual cost” will be calculated for each type of equipment based on the acquisition cost of the equipment, the depreciation period and the discount rate. Once we have obtained these equivalent annual costs, we will choose the relevant unit of production in order to obtain an equipment operation cost for each analysis: for equipment entirely dedicated to the intervention being considered, we will divide the equivalent annual cost by the number of interventions carried out in the year; for shared equipment, we can use a pro rata of the equipment operation time for the intervention being considered.

An average cost of analyzing a sample will be arrived at after adding a proportion attributable to the institution's overheads. A sensitivity analysis will document the variations in the mean total costs in each group depending on the variation of a number of assumptions: life cycle of the equipment, discount rate, acquisition price of the equipment. Lastly, different levels of dissemination of the technique and consequently different levels of annual activity can be simulated: the consequences on the cost of operating the equipment, and consequently on the average cost of the examination at constant staffing levels (economies of scale allowed) can be taken into account.

d. Other unit costs

Other cost factors will be costed based on the unit costs at our disposal in the healthcare system: national nomenclatures (common classification of medical acts or CCAM, nomenclature for medical laboratory services or NBAM), market prices, etc. With regard specifically to hospital stays, a daily cost will be calculated on the basis of ENCC (national cost survey with common methodology) data and length of stay associated with specified DRG for each patient in our study.

e. Incremental cost-effectiveness ratio

The average cost of implementing the screen-and-treat strategy as well as the cost of standard care over the 6 months of follow-up covering the pregnancy period will be identified. An incremental cost between the two care groups will be calculated.

The difference in effectiveness will be calculated by subtracting the rate of preterm delivery before 37 weeks' gestation between the Group B and Group A.

The incremental cost will be divided by the difference in effectiveness and this incremental cost-effectiveness ratio will correspond to the additional cost per additional avoided preterm birth before 37 weeks.

This ratio will be reflected at each stage of preterm delivery: before 26-28 weeks' gestation, before 32 weeks' gestation, and before 36 weeks' gestation.

f. Acceptability threshold (A SUPPRIMER)

g. Additional analysis: indirect costs

The duration and causes of work loss will be analyzed and the cost of work loss outside periods of maternity leave can be further assessed, if linked to the strategy under review.

These will be collected by the Short Form Health and Labor Questionnaire (SF-HLQ) (Van Roijen 1996), a validated generic instrument consisting of 11 questions, used to collect information on production losses related to health problems in individuals who are paid for their labor, as well as their quality of life outside work.

They will be valued based on a method known as "friction cost" (Koopmanschap 1995).

4. **Budget impact analysis**

A budget impact analysis will be carried out at national level from the payer's point of view in order to inform public decision-making (CES 2008). It will be based on simulating the translation of the outcomes observed under experimental conditions into what can be expected amongst the population of women concerned by the innovation in real conditions, across the country. A measure of the budgetary impact of adopting the innovation will be proposed and presented for different assumptions.

The parameters for this model will consist of the hypotheses about the levels of screening uptake (effective dissemination) and the costs measured in the cost-effectiveness analysis.

Our approach will be based on a methodology summarized in the figure in Appendix 5.

An inventory will be carried out by interviewing experts and medical and institutional operators involved in the implementation of this innovation (via a questionnaire developed for

the occasion). Questions will include:

- the current size of the target population;
- And the size of the population reached out to for BV screening (epidemiological data from the facilities/departments concerned).

The diagnostic and therapeutic regimens, including innovation, the measurement of their implementation, induced and avoided costs and their consequences, will be derived from the results of the cost-effectiveness analysis.

A questionnaire will be drawn up and administered to the expert, medical and institutional stakeholders involved in the dissemination of this innovation. Questions will include:

- the possible extension of the indication (modified size of the target population);
- the anticipated volume of patients who could benefit from the innovation (modified size of the population sought);
- the feasibility of deploying the POC technique in their area of activity and across the country;
- the incentive impact of pricing for this activity assessed from different pricing levels;
- the changes in medical strategies linked to the dissemination of the innovation: degree and conditions for substituting it to the standard strategy, induced effects, etc.

All these questions will be based on a 3-year timeframe, in order to allow the dissemination cycle of the innovation to be completed.

The budget impact model will be presented in a multi-sheet spreadsheet format. The baseline parameters expressing different hypotheses (regarding induced/avoided costs, stakeholder behavior, etc.) can be modified in order to measure the robustness of the results to the variations (univariate and multivariate sensitivity analyses) or to develop other simulation scenarios.

F. Sample size and justification

The primary endpoint is the incremental cost-effectiveness ratio. Based on the methodology proposed by Briggs et al. for calculating the number of subjects needed in a cost-effectiveness analysis (Briggs 1998) and based on the following hypotheses:

- effectiveness difference in terms of percent of preterm births before 37 weeks' gestation being 0.013, based on a preterm delivery rate of 0.043 in Group B and 0.03 in Group A;
- average cost difference being €230 +/- 35. This difference takes into account the estimated cost of POC technique (initial and recurrence) and the estimated cost of treatment for BV (10% of patients);
- the ICER threshold set at €22,500, corresponding to the average avoided cost of caring for a child born prematurely before 37 weeks' gestation as documented in literature (Petrou 2012);

with an 80 % statistical power and a threshold for statistical significance set at 0.05, 2,961 subjects are needed per group. Assuming a 15% drop-out rate, a total of about 6,800 subjects should be included.

The recruitment will be carried out in 1 year (12 months of inclusion), while accounting for the current patient workload and the recruitment capacities of the 20 participating centers. The expected recruitment per center (as well as the patient workload population) is presented in the table below.

**Centers (patient population)**	**Recruitment**
**South Zone**	
AP-HM (Public academic teaching hospital La Conception - 3500 deliveries; Public academic teaching hospital Nord - 2900; Périnat Sud Network 44,000)	3000
Saint Joseph Private Hospital - Marseille	150
Public academic hospital, Aix-en-Provence	150
Bouchard Private Hospital- Marseille	50
Public hospital, Aubagne	100
Public academic hospital, Toulon	150
Public academic teaching hospital, Nice	300
Public academic teaching hospital, Nîmes (2200)	200
Public academic teaching hospital, Saint Etienne	150
Public hospital, Pau	50
Public academic teaching hospital, Guadeloupe	50
Public academic teaching hospital, Martinique	50
**North Zone**	
Public General Hospital, Poissy (4300)	500
Public academic teaching hospital Kremlin Bicêtre, Kremlin Bicêtre (2500)	300
Public academic teaching hospital Robert Debré, Paris (3200)	300
Public academic teaching hospital, Clamart (2545)	200
Maternal and Infant Protection Unit of the Conseil Général du Val de Marne	300
Public academic hospital, Créteil	300
Public academic teaching hospital, Angers (2600)	300
Public academic teaching hospital Armand Trousseau, Paris	200
**Total**	**6800**

G. Participation period

The duration of inclusion has been scheduled to be 30 months. Each subject will be monitored for a period of 12 months (6 months of pregnancy until term and 6 months post-term). The duration of the study is 48 months.

IV. Research design and conduct

A. Division of labor

1. Research Steering Committee

A steering committee will be set up including the investigating physicians, the coordinating clinical study technician, the methodologist and the health economists.

It will be responsible for setting up the e-CRF and ensuring the smooth running of the study.

It will be convened thrice:

1/ before the start of the study to approve the various documents such as the CRF, the information leaflet and the consent form;

2/ 6 months after the start of the study to check the number of subjects included, drop-outs and to identify any dysfunctions;

3/ at the end of the study to validate the results and organize the scientific publication of the data.

2. Scientific Committee

The Scientific Committee is made up of national experts in the field of obstetrics, and more specifically in the field of BV. The role of this Committee is to participate in the interpretation of the scientific outcomes of the study and to provide a critical analysis before publication and dissemination. The Scientific Committee will be comprised of Prof. D'Ercole, Prof. Goffinet and Prof. Subtil.

3. Gynecology-Obstetrics departments of participating centers

The investigating physicians and midwives associated with the project will be responsible for recruiting and enrolling patients, ensuring that vaginal self-sampling is carried out, in addition to collecting clinical data. At the inclusion consultation, the doctor or midwife at the center will include the patient and randomize her on the CleanWeb software.

The two centers will be in charge of organizing and setting up the POC in the various centers and the molecular analysis of the samples.

The results will be sent by the POC laboratory by email or fax or telephone within 12 hours of inclusion to the physician and/or midwife, as well as to the clinical study technician coordinating the project (see chapter on managing positive results).

The clinical study technician coordinating the project reports to the principal investigator and the Clinical Investigation Centre of Nord Hospital.

Management of patients with positive swabs:

The positivity of the result is defined based on molecular biology techniques only and in accordance with prior publications (Menard 2008, PHRC National 2006). Evidence of *Atopobium vaginae* $\geq 10^8$ copies/mL and/or *Gardnerella vaginalis* $\geq 10^9$ copies/mL defines bacterial vaginosis (BV).

The clinical study technician coordinating the project will be in charge of calling for control swabs from patients with a positive sample in coordination with the team that included the patient. They will be offered a consultation with the midwife and/or doctor, and treatment for

those with a positive result will be started as soon as possible and within a maximum of 24 to 48 hours after inclusion. Treatment with azithromycin and amoxicillin in case of failure or allergy shall be supplied to the centers by the study. The midwife and/or doctor at the center will give the patient a swab kit to take a control vaginal swab. Depending on the organization of each inclusion center, adjustments to the results delivery scheme may be proposed while maintaining rapid result delivery.

An alert for controls and term of delivery will be created on CleanWeb.

4. **Role of the midwives**

At each inclusion center, midwife involvement time is assigned according to the number of scheduled inclusions. Midwives will carry out inclusion after ensuring informed consent, randomization in CleanWeb, and will fill in the electronic logbooks. In the event of a positive result, the midwives will be in charge of, in conjunction with the coordinating clinical study technician, summoning the patient and/or sending her the treatment in accordance with the protocol, having ensured absence of allergy. They will also check that the patient has understood the need for the efficacy control at day 15 and subsequent controls.

5. **Methodological Support Unit for Clinical Research and Medico-Economic Evaluation**

The economist associated with the study will provide permanent support to the principal investigator in all stages of the study: project drafting, preparing documents to obtain authorizations (in collaboration with the AP-HM Clinical Research Department), designing the case report forms, study management, data analysis, as well as drafting the final report and scientific publications. The methodologist physician and the statistician will be mainly involved in the analysis of the clinical data, drafting of the final report and the scientific publications, as well as participation in the drafting of the project.

6. **The Clinical Research and Innovation Department of the AP-HM**

The AP-HM's Clinical Research and Innovation Department will be responsible for the administrative and financial follow-up of the study, in addition to ensuring the necessary authorizations are obtained to conduct the study, serving as the main contact for links with the other participating establishments as well as organizing the quality control of the data collected in accordance with Good Clinical Practice.

B. Follow-up organization

1. Inclusion consultation

The investigating physician or the midwife associated with the project will conduct the interview and clinical examination of the patient. They will verify all the selection criteria. They will explain to the patient the objectives of the study, as well as the process, advantages and disadvantages of participating. They will obtain the patient’s informed consent and then randomize the patient.

For Group A patients, vaginal self-sampling will be performed at the inclusion visit. Depending on the patient's choice or in case of difficulties, the sample can be taken under speculum. Vaginal self-sampling with a cotton swab on a dry tube will be performed by the patient after prior instruction by the doctor or midwife and after reading the vaginal self-

sampling method. A similar illustration has already been published by Bresson (2006) and has been developed to explain this sampling technique in simple terms.

2. **Bacteriological analysis**

a. Conventional processing of vaginal swabs in the laboratory

In low-risk populations, in the absence of symptoms, vaginal swabbing is not recommended.

However, if a vaginal swab is taken, the results (Nugent score) will be retrieved and the treatment incorporated into the analysis.

b. Quantification by real-time PCR

The molecular quantification tool will allow the quantification of *A. vaginae*, *G. vaginalis* and human albumin from the vaginal swab. The quantification of microorganisms will be based on the specific real-time PCR technique associated with a dilution of a quantification plasmid.

The protocol is as follows:

- **DNA extraction**

On arrival at the laboratory, the vaginal swab is discharged into 600 µl of BME (Basal Medium Eagle, Gibco). After suspending the sample in BME, 500 µl will be stored at -20°C for later analysis and 100 µl will be taken, added to proteinase K (12 µl) and Buffer G2 (138 µl; EZ1 DNA tissue Kit, QIAGEN), vortexed and incubated in a dry bath for 10 minutes at 70°C. After incubation, 200 µl of the latter suspension was taken in order to perform a DNA extraction for 18 minutes on the BioRobot EZ1 automated system (QIAGEN).

- **Quantitative real-time PCR**

Strips with wells containing the reagents (mix, primers, probe, DNASE water and RNASE free) necessary to perform the *G. vaginalis*, *A. vaginae* and human albumin PCRs, the synthetic positive control (dilution to 10^4 of a quantification plasmid) and the negative control (sterile water for the reagent control) are prepared in advance and stored at -20°C. Finally, 5µl of the vaginal sample DNA extract pure and diluted to 1/10 will be placed in the wells for quantitative real-time PCR targeting *G. vaginalis*, *A. vaginae* and human albumin. The remaining DNA extract will be stored at -20°C for further analysis. These quantitative real-time PCRs will be performed on the Bio-Rad CFX machine and the results are available after an amplification protocol of 1 hour and 20 minutes. The interpretation of the data is carried out after validation of the quality of the positive and negative controls. The presence of *A. vaginae* (> 10^5 copies/mL) and/or *G. vaginalis* (> 10^5 copies/mL) will indicate presence of the risk of preterm birth. The BV diagnosis will be made when BV has been defined as *A. vaginae* $\geq 10^8$ copies/mL and/or a *G. vaginalis* load $\geq 10^9$ copies/mL and in this case treatment will be initiated.

Microbial quantification is considered for each sample if:

1) for the 10^4 dilution of the quantification plasmid, the Ct (cycle threshold) value is 30;

2) the Ct (cycle threshold) values for each tested pure and diluted microorganism are

reproducible and linear;

3) the variation in albumin copy number, used as an internal control, is narrow and the albumin quantification values must be greater than 10^2 copies per 5µl of DNA extract.

The threshold of positivity for the quantification of microorganisms is 10 copies per 5µl of DNA extract. The results of the microbial quantification will be expressed as DNA copies for each microorganism per 1 milliliter of vaginal secretion suspension.

The DNA samples and extracts taken during the study will be stored as a biological collection. The head of the collection will be Dr Florence Fenollar. The samples will be kept at the Rickettsia Unit (Méditerranée Infection Foundation). The samples will be kept after the research is completed. The collection will be declared to the competent authorities. Patients will always have the option of withdrawing or objecting to retention.

3. **Therapeutic management and vaginal self-sampling**

If the result is positive, the patient will be contacted by telephone by the midwife or doctor at her center and told the result (D0). A consultation with the midwife and/or doctor will be offered within 48 hours, ideally on the same day. The treatment with azithromycin 2 g in total in 2 doses - at D1 (1 g) and at D3 (1 g) - will be given to the patient. In case of contraindication to azithromycin, a treatment with amoxicillin 2 g per day for 7 days will be proposed.

A first control sample at D18 (15 days after treatment) by self-sampling will be scheduled. Two further control samples will be taken by self-sampling at D48 and D78.

The 3 home sampling kits (cotton swab, instructions for use and a questionnaire) will be given to the patient at the time of the result consultation. The patient will also be monitored as usual. In case of difficulty, these samples can be taken during the consultation upon request.

These samples will be sent in pre-stamped envelopes to the bacteriology laboratory in Marseille and to the POC center of the molecular biology laboratory at the Poissy St Germain Hospital.

The practitioner in charge of the patient's follow-up or the midwife will be informed by email of the results of the molecular analysis by the coordinating CRA.

4. **Management of treatment failure and recurrence of vaginal flora anomalies**

Cure following treatment will be defined as a negative molecular test at the D18 control sample (15 days after treatment): *A. vaginae* load < 10^8 copies/mL and/or *G. vaginalis* load <10^9 copies/mL.

Failure of first-line treatment will be defined as a positive molecular test at the D18 control swab (15 days after treatment). In this case, in the absence of allergy, treatment with amoxicillin 2 g per day for 7 days will be initiated.

Failure of the second-line treatment will be defined as a positive molecular test at the D48 control swab. In this case, a new (third-line) treatment will be proposed; it will be chosen based on discussions between the investigators and the principal investigator of the study.

Failure of third-line treatment will be defined as a positive molecular test at the D78 control swab. The relevance of a fourth-line treatment will be discussed between the investigators and the principal investigator of the study, especially depending on the term of pregnancy of the patient concerned.

Recurrence of vaginal flora imbalance will be defined according to the same criteria defined above after being cured (negative result at D18). It can be detected at the D48 or D78 control samples.

In the event of a diagnosed recurrence, treatment with azithromycin 2 g in total in 2 doses on D1 (1 g) and D3 (1 g) will be repeated. If azithromycin is contraindicated, amoxicillin 2 g per day for 7 days will be repeated.

Treatment failure or cure of the recurrence can be determined at the D78 control sample. In case of failure, the relevance of a new treatment will be discussed between the investigators and the principal investigator of the study, especially depending on the term of pregnancy of the patient concerned.

In case of vaginal infections other than a recurrence of vaginal flora imbalance, the treatment of patients will be adapted to the infection according to current recommendations (WHO 2005, Menard EMC 2012).

5. Study exit

The study exit will take place 6 months after delivery.

V. Data processing

A. Data collection

1. Organization

Inclusion data will be collected by the investigating physician and/or the dedicated midwife during the inclusion visit.
The mobile coordinating clinical study technician will be in charge of retrieving pregnancy follow-up data, delivery data and the outcomes of patients and their newborns 6 months after delivery. Patients will be contacted by telephone and the data to be recorded in the case report form will be collected both by oral questioning and by consulting their medical records. In the event of hospitalization, the reports will be retrieved to ensure the reliability of the data.

Data on quality of life and follow-up of work stoppages will be collected by means of questionnaires to be completed:

- directly by the mothers during the inclusion visit;
- and by questioning them orally during the systematized telephone call upon study exit (6 months).

The data collected in this manner will be recorded in an electronic case report form developed in the CleanWeb software.

2. Data collected at inclusion

For all included patients, the data collected at inclusion were:

- Socio-demographic data: age, ethnic origin, family situation (living with a partner, number of dependent children), education level, socio-professional category.

3. Data collected at the time of delivery

Group A:

- Biological data: number of samples and analyses, molecular test results and characteristics of the molecular analysis for each sample (number and type of manipulations to obtain the result of the initial diagnosis and that of the recurrences).
- Data on antibiotic treatment(s) in pregnancy in connection with the protocol and treatment prescribed outside the protocol with azithromycin and/or amoxicillin and/or probiotics: effectiveness, side effects.
- Data on the evolution of the symptomatology.
- Data on treatment failure and recurrence.

Group B:

- Data on potential screening for BV: number and type of tests; results; possible screening for recurrence.
- Data on therapeutic management in case of a BV diagnosis: type of treatment, duration, effectiveness, side effects.
- Data on the evolution of the symptomatology.
- Data on treatment failure and recurrence.

In the 2 groups:

- Data on pregnancy complications: premature rupture of membranes, risk of preterm birth, spontaneous abortion.
- Term and delivery modalities; birth weight, Apgar score.
- Existence of endometritis (hyperthermia >38°C associated with suspicious loci and pelvic pain and a C-reactive protein test >20 mg/L).
- Lab tests (CBC, CRP, blood culture if necessary, vaginal swab).
- Data on treatment during pregnancy: oral or subcutaneous treatment, antibiotic, antifungal or antiparasitic treatment.
- Data on the use of the healthcare system during pregnancy: number and types of hospital stays, number of consultations, emergency admissions, number of complementary tests.

4. Data collected at the end of the study

- Data on the use of the healthcare system in the 6 months postpartum for the baby and the mother: number and types of hospital stays, number of consultations, emergency admissions, number of complementary tests.
- Neonatal morbidity data;
- Neonatal mortality data;
- Self-administered data: quality of life (SF-12), professional situation and work stoppages (SF-HLQ), consumption data at the patient's expense (home care, transport, etc.).

5. Data collected before the study was set up and at the end of the study

Questionnaires drawn up by the economist in association with the principal investigator will be sent to the investigators as well as to institutional stakeholders (hospitals, French National Health Authority-HAS, National Health Insurance Fund, etc.) in order to analyze the budgetary impact of the innovation. Details provided in Part IV. E. 4.

B. Quality control

A clinical research associate will be responsible for checking the content and filling in the case report forms as inclusions occur. He will also check that informed consent has been obtained from the patient in accordance with regulatory guidelines. Particular attention will be paid to the collection of the primary endpoint.

C. Data entry

All information required by the protocol must be provided and an explanation given for each missing element. Data should be transferred to the computerized e-CRFs as they are obtained, whether clinical or paraclinical data. The CleanWeb software will be installed in the participating centers. The software will be used for inclusion, randomization and data collection.

D. Statistical analysis of data

The main principles of the analysis are reported below. However, a more detailed specific analysis protocol will be drafted and submitted for validation (coordinating investigator, associated investigators, analysis manager and biostatistician).

Statistical use will only begin after the validity of the database has been verified (issue of requests to the clinicians involved in the study, consistency checks). There is a procedure and an algorithm for rendering data anonymous that assigns a number to each individual. A correlation table will be available, separate from the operating base. Only the number will be entered into the computer database. The database will then be locked. After locking the database, the consolidated data will be processed by the statistician. Data analysis will be carried out using SPSS version 17.0 software in Windows, by the statistician of the Clinical Research Methodology Unit, Clinical Research Department Marseille (headed by Prof. Pascal Auquier) and the economists involved in the study. The significance threshold for the interpretation of the tests is set at 0.05.

1. Test populations

Statistical analysis will be performed on the intention-to-treat population (main analysis), including patients for whom a major protocol violation is observed (no objective post-inclusion data, wrongly included patient). Additional per-protocol population analysis (secondary analysis) will be performed if necessary. No interim analysis has been scheduled.

2. Population description, initial comparability of the groups

First, a descriptive analysis of the entire sample will be carried out. Qualitative variables will be presented as proportions and numbers, quantitative variables as mean and standard deviation, or median and quartiles. For each variable, the proportion of missing data will be specified. The normality of the parameters will be assessed using frequency histograms and

Shapiro tests; simple mathematical transformations can be used to normalize non-normal data.

The comparability of the 2 groups ("standard strategy" and "innovative strategy") will be carried out on all the variables available at inclusion in order to ensure initial comparability: using chi-square tests for qualitative variables, and Anova tests for quantitative variables. The center variable will be considered as a random variable in order to account for the expected imbalance in the numbers included per center.

3. **Analysis of the primary endpoint: incremental cost-effectiveness ratio and addressing uncertainty**

An incremental cost-effectiveness ratio expressed as the cost per avoided preterm delivery before 37 weeks' gestation will be calculated and presented with its 95% confidence interval, calculated based on the truncated Fieller method (Siani 2006; De Peretti 2006), in order to account for the uncertainty related to sampling fluctuations. A graphical representation based on nonparametric bootstrapping, as recommended by the French National Health Authority (HAS 2011), will be proposed. To this end, 10,000 simulated bootstrap samples will be generated by means of independent draws, with a discount from the pairs constituted by the difference in average costs and the difference in average efficiencies between the two treatments compared in such a way that the correlation between cost and efficiency is preserved. These 10,000 pairs will be represented by a scatter plot corresponding to an estimate of their joint distribution (Claxton 2005). The confidence intervals of the pairs will be represented by ellipses: an outer ellipse corresponding to the 95% confidence interval of the pair and an inner ellipse corresponding to the 50% confidence interval of the pair (French National Healthcare Authority-HAS 2011). For further robustness, in addition to the confidence ellipses described and provided, the uncertainty around the incremental cost-effectiveness ratio will be accounted for by calculating the probability that it belongs to each quadrant of the cost-effectiveness plane.

In order to take into account the uncertainty linked to the assumptions made on the model parameters and to test the robustness of the outcomes, univariate or multivariate deterministic sensitivity analyses will be carried out on the different key parameters of the analysis and will be subject to a Tornado diagram.

4. **Analysis of secondary endpoints**

The comparison of the 2 groups based on binary-type secondary endpoints (preterm birth rate before 37 weeks' gestation, corrected spontaneous preterm birth rate, respiratory syndrome rate, bronchopulmonary dysplasia, intraventricular hemorrhage, leukomalacia, etc.) will be carried out using a chi-square test and that of the quantitative variables (overall hospitalization, before birth, after birth, etc.) will be carried out using Student's t-test or Mann-Whitney U test.

In Group A, "innovative strategy", the proportions of recurrence and treatment success will be documented and presented with their 95% confidence intervals; changes in microbial concentrations before treatment and 1 month after treatment will be tested using paired t-tests. In this group, the time between sampling and delivery will be estimated by the Kaplan-Meier method, and this time will be compared between groups of vaginal concentrations of the different pathogens using the log-rank test (the cut-offs defining pathological vaginal concentrations are defined by the outcomes of the National Clinical Research Program (PHRC): *A. vaginae* $\geq 10^8$ copies/mL and *G. vaginalis* $\geq 10^9$ copies/mL).

In the "Standard" Group B, the proportions of patients who received Nugent screening will be documented, as well as the proportions of positive results, treatment initiated, and treatment effectiveness. The results will be produced with their 95% confidence intervals. This subgroup of patients will be subject to calculation of incremental cost-effectiveness ratios in comparison with the innovative strategy on the one hand, and with the standard strategy on the other hand.

The total costs will be presented as an average per patient for each group; the relative burden of each of the cost factors in the total cost will be identified.

The costs will be subject to statistical processing in the same manner as the other quantitative variables (mean comparison using Student's or Mann-Whitney tests). Generalized linear models (log or gamma type) could be contemplated in order to explain the "average total cost per patient" variable based on a selection of explanatory variables.

The scores of the dimensions of the quality of life questionnaires will be calculated from the algorithms provided by the developers. The scores of the dimensions at 6 months will be compared between the 2 groups in relation to the initial score (t-tests or Mann-Whitney U test).

In order to account for the expected imbalance in the number of inclusions per center, as well as the differences in care that may exist between centers, the center effect will be controlled. Literature reviews per center will complement the analyses of the overall population.

5. Multivariate analyses

Multivariate analyses will be performed using logistic regression models. The variable to be explained will be represented by the 37 weeks' preterm birth yes/no variable; the selection of the explanatory variables will be based, on the one hand, on the univariate approach which will identify variables for which the *P* value is lower than or equal to 0.20 (the group variable will be automatically selected), and, on the other hand, on the prior identification of variables potentially associated with preterm birth. The results will be presented in the form of odds ratios and their confidence intervals.

E. Management of serious adverse events (SAEs)

Vaginal swabs are a common practice during pregnancy. Thus, no serious adverse events are expected from physician vaginal swabbing or from vaginal self-sampling during and outside of pregnancy.

VIII. Total expected duration of the research

The total duration of the study is 48 months (M) including 12 months of inclusion.

Estimated study start date: October 2014.

M-4 – M0: Preliminary stage: preparation of case report forms, submission to the Ethics Review Board and ANSM (French authority in charge of pharmaceutical and health product safety)

M1 – M30: inclusion of patients 30 months

31 – 42: patient follow-up (6 months)

42 – 48: quality control, analysis, report writing

IX. Expected outcomes – Prospects

The expected outcomes are related to the reduction of preterm birth thanks to the treatment-prevention of BV and its recurrence: if this screening reduces the preterm birth rate by 1.3%, we estimate that approximately 10,400 preterm births would be avoided per year. In our study of 3400 patients screened using molecular testing compared to the unscreened group, **we estimate that we can avoid about 40 preterm deliveries in the screened group and realize substantial cost-savings related to the avoided management of the preterm births**.

Prospects: Screening for flora anomalies in early pregnancy could be offered systematically in the population and would contribute to the reduction of preterm birth.

X. Clinical trial vigilance

A. Definitions

1. Adverse event (AE)

Any adverse event in a person undergoing biomedical research, whether or not the event is related to the research or to the experimental drug(s) upon which this research is based.

2. Serious adverse event (SAE)

A serious adverse event is an event:

- whose progress is fatal,
- or which endangers the life of a person participating in the research,
- or which results in a significant or lasting disability or handicap,
- or which results in initial or prolonged hospitalization,
- or which results in a congenital anomaly or malformation,
- or any other event that does not meet the qualifications listed above but can be considered as "potentially serious",
- or medically relevant event as determined by the investigator,
- or an event requiring medical intervention to prevent progression to one of the above conditions.

The expected serious adverse events in the research and which are considered as adverse events in this protocol are as follows:

- ➢ serious side effects mentioned in the SPC (summary of product characteristics) of azithromycin and amoxicillin.
- ➢ expected obstetric complications:

- Hypertension
- Gestational diabetes
- Fetal growth restriction

- Preeclampsia
- HELLP syndrome
- Retroplacental hematoma
- Placenta Previa
- Risk of preterm birth
- Premature rupture of the membranes
- Chorioamnionitis
- Risk of late miscarriage

These expected serious adverse events will not be reported to the sponsor, but will be recorded in the case report forms (as adverse events).

3. **Adverse reaction to an experimental drug (AE)**

Any harmful and unwanted reaction to an experimental drug at any dose.

4. **Unexpected adverse reaction**

Any adverse reaction to the experimental drug whose nature, severity or development is not consistent with the information contained in the reference document: Summary of Product Characteristics or Investigator's Brochure.

B. Investigator's responsibilities

1. **Procedures for detecting and documenting adverse events**

All adverse events should be investigated, reported and recorded, processed and evaluated from the first visit (D0 inclusion) until the end of the study and their resolution.

2. **Reporting of serious adverse events**

The investigator assesses each adverse event in terms of its severity.

The investigator should notify the sponsor within 24 hours of becoming aware of all serious adverse events in the trial, except for listed obstetrical complications which are not reportable to the sponsor, but should be recorded in the case report forms as an adverse event.

The investigator should document the event to the best of his/her ability, provide a **medical diagnosis** if possible, and establish a causal link between the serious adverse event and the experimental drug(s) and/or associated treatment(s) and/or the research.

The report should be sent to the sponsor using a signed and dated Serious Adverse Event Report **Form** attached to the case report form, together with copies of the laboratory results or test reports or hospitalization reports documenting the serious event, including relevant negative results, **without omitting to anonymize the documents** and to enter the patient number and code.

The investigator should ensure that relevant follow-up information is provided to the sponsor within 8 days of the first report.

The investigator should monitor the patient who has experienced a serious adverse event until resolution, stabilization at a level acceptable to the investigator, or return to baseline, even if the patient has been discharged from the trial, and inform the sponsor of the progress of the serious adverse event.

Notification can be made by fax or email to the sponsor using the Serious Adverse Event Report Form, which is available in the case report form and investigator's folder, by submitting it to:

Clinical Research and Innovation Department of the AP-HM<br>80, rue Brochier, 13354 Marseille Cedex 05<br>Telephone: +33 (0)4 91 38 27 47. Fax: +33 (0)4 91 38 14 79<br>Email: dir.recherche@ap-hm.fr

3. **Assessment of causality**

The investigator should assess the causal relationship of serious adverse events to the experimental drug(s), comparator(s), any associated treatment(s) and the research. All serious adverse events to which the investigator or sponsor believes that a causal relationship can be reasonably attributed are considered as suspected serious adverse events.

4. **Reporting period**

All serious adverse events must be reported, if they occur for a research participant:

- From the date of signing the consent form,
- For the duration of the participant's follow-up in the trial,
- Up to 4 weeks after the end of the trial for the research participant.

C. Sponsor's responsibilities

1. **Reporting of unexpected serious adverse reactions**

The sponsor should assess the causal relationship between the serious adverse event and the experimental drug(s) and associated treatments and the research.

The sponsor assesses whether the adverse reaction is expected or unexpected guided by the reference document (side effects mentioned in the Summary of Product Characteristics of azithromycin and amoxicillin can be considered as expected).

The sponsor reports any serious and unexpected adverse events to the EMA (EudraVigilance, European pharmacovigilance database), to the competent health authorities and to the relevant Ethics Committees within the regulatory deadlines and informs the investigators at intervals appropriate to the research.

The regulatory report shall be made within a maximum period of:

- 7 calendar days for serious unexpected life-threatening or fatal adverse events. In such cases, additional relevant information must be sought and provided within a further 8 days.

- 15 calendar days for all other serious unexpected effects. Similarly, additional relevant information must be sought and provided within a further 8 days.

In the case of a masked trial, as a general rule, the sponsor reports the serious unexpected adverse reaction to the relevant health authorities and Ethics Committees after unmasking the experimental drug.

2. **Safety developments reporting**

The sponsor also reports any safety developments to the relevant health authorities and Ethics Committees and sends them an annual safety report.

3. **Annual safety report**

On the anniversary date of the trial authorization issued by the Health Authorities, the sponsor draws up a safety report including:

- A list of serious adverse events that may be related to the experimental drug(s) in the trial, including unexpected and expected serious events.
- A concise and critical analysis of patient safety for research purposes.

The report may be submitted to the coordinating investigator for approval and is sent to the relevant Health Authorities and Ethics Committees within 60 days of the anniversary date of the trial authorization.

4. **Independent monitoring committee**

There is no need for an independent monitoring committee for this research due to the low risk to patients.

XI. Publication rules

The recruiting centers will participate in the publications resulting from this study. The order will depend on the investment in the project, the number of inclusions and the scientific contribution.

XII. Legal and ethical aspects

This research will be conducted in accordance with the Declaration of Helsinki (World Medical Association), which sets out the ethical principles applicable to medical research on human subjects, and in accordance with the Guide to Good Clinical Practice, which allows for international harmonization of the conduct of human trials in accordance with the different legislations.

The prospective sponsor of this project is represented by the Assistance Publique des Hôpitaux de Marseille (AP-HM). A regulatory watch will be carried out by the sponsor. The sponsor will submit the project to the relevant authorities for approval.

This project is part of a biomedical interventional research, as defined in Article L.1121-1, regarding a product mentioned in Article L.5311-1 of the French Public Health Code (drug); it is subject to the new regulations that apply to research "organized and carried out on human beings with a view to developing biological and medical knowledge", namely, the Public Health Act no. 2004-806 dated August 9, 2004 pertaining to public health policy and its

application decrees dated August 27, 2006, aimed at aligning French regulations with European law. As such, it will be subject to a request for a favorable opinion from a Committee for the Protection of Individuals, and a request for authorization from the Competent Authority represented by the French National Agency for the Safety of Medicines and Health Products (ANSM).

An information leaflet will be distributed to patients and informed consent will be obtained. They will be written in accordance with regulatory recommendations, including the purpose of the study, the benefits and risks associated with the study, how the study will be carried out and all legal provisions to which patients are entitled. This research will be conducted in accordance with Good Clinical Practice, which is a set of ethical and scientific quality requirements that must be respected in the planning, implementation, conduct, monitoring, quality control, auditing, data collection, analysis and reporting of outcomes Adhering to the Good Clinical Practices ensures protection of rights, safety and protection of the individuals who undergo the research and the preservation of their anonymity, as well as the credibility (integrity, authenticity, verifiability) and accuracy of the data and outcomes of the research.

There will be no alterations or changes to this protocol without the agreement of all investigators. Any amendments to the study protocol should be notified to the Ethics Review Board if the planned alterations modify the ethical or medical/scientific aspects of the study. The investigators undertake to respect the legislative obligations in force and to conduct this study in accordance with Good Clinical Practice. The protocols and case report forms will be handed over during the startup visit to the center by the CRA. Information collected from patients will be kept strictly confidential. They will be kept in paper format inside locked premises. They will be input into a computer and will be processed automatically.

This computerized processing will not make it possible to directly or indirectly identify the subjects. All of these data will be accessible only to the principal investigator and the sponsor's representatives, or to the authorized health authorities if necessary. A declaration of the study will be made to the French Data Protection Authority in accordance with the legislation in force (Data Protection Act of January 6, 1978, amended by the Act dated July 1, 1994 and the Decree dated May 9, 1995). The participating subject must be informed of the nature of the information processed, its purpose, and the identity of the natural and legal persons receiving the data. The participant will retain the right to access and rectify this data through a doctor of her choice, as well as the right to object in accordance with European Directive 95/46/EC. In accordance with the Act dated March 4, 2002 on patients' rights and the quality of the health system, the overall outcomes of the study may be communicated to the subjects at their request, either directly or through a doctor of their choice.

XIII. References

Amsel R, Totten PA, Spiegel CA, Chen KC, Eschenbach D, Holmes KK. Nonspecific vaginitis. Diagnostic criteria and microbial and epidemiologic associations. Am J Med 1983;74:14-22.

Andrews WW, Hauth JC, Goldenberg RL. Infection and preterm birth. American Journal of Perinatology. 2000;17(7):357–365.

Anhang R, Nelson JA, Telerant R, Chiasson MA, Wright TC Jr. Acceptability of self-collection of specimens for HPV DNA testing in an urban population. J Womens Health 2005;14:721-8.

Aroutcheva AA, Simoes JA, Behbakht K, Faro S. Gardnerella vaginalis isolated from patients with bacterial vaginosis and from patients with healthy vaginal ecosystems. Clin Infect Dis 2001;33:1022-7.

Baay MF, Verhoeven V, Lambrechts HA, Pattyn GG, Lardon F, Van Royen P, Vermorken JB. Feasibility of collecting self-sampled vaginal swabs by mail: quantity and quality of genomic DNA. Eur J Clin Microbiol Infect Dis 2009;28:1285-9.

Beaino G, Khoshnood B, Kaminski M, Marret S, Pierrat V, Vieux R, Thiriez G, Matis J, Picaud JC, Rozé JC, Alberge C, Larroque B, Bréart G, Ancel PY; EPIPAGE Study Group Predictors of the risk of cognitive deficiency in very preterm infants: the EPIPAGE prospective cohort. Acta Paediatr. 2011 Mar;100(3):370-8.

Beigi RH, Austin MN, Meyn LA, Krohn MA, Hillier SL. Antimicrobial resistance associated with the treatment of bacterial vaginosis. Am J Obstet Gynecol 2004;191:1124-9.

Berbis J, Einaudi MA, Simeoni MC, Brévaut-Malaty V, Auquier P, D'Ercole C, Gire C. Quality of life of early school-age French children born preterm: A cohort study. Eur J Obstet Gynecol Reprod Biol. 2012;162(1):38–44. doi: 10.1016/j.ejogrb.2012.02.006.

Blondel B, Lelong N, Kermarrec M, Goffinet F; National Coordination of Perinatal National Surveys. Trends in perinatal health in France between 1995 and 2010: Results from the National Perinatal Surveys.J Gynecol Obstet Biol Reprod (Paris). 2012 Apr;41(2):151-66.

Bothuyne-Queste E, Hannebicque-Montaigne K, Canis F, Noulard MN, Plennevaux JL, Tilloy E, Subtil D. Is the bacterial vaginosis risk factor of prematurity? Study of a cohort of 1336 patients in the hospital of Arras. J Gynecol Obstet Biol Reprod (Paris). 2012 May;41(3):262-70.

Bradshaw CS, Morton AN, Hocking J, Garland SM, Morris MB, Moss LM, Horvath LB, Kuzevska I, Fairley CK. High recurrence rates of bacterial vaginosis over the course of 12 months after oral metronidazole therapy and factors associated with recurrence. J Infect Dis 2006;193:1478-86.

Bradshaw CS, Tabrizi SN, Fairley CK, Morton AN, Rudland E, Garland SM. The association of *Atopobium vaginae* and *Gardnerella vaginalis* with bacterial vaginosis and recurrence after oral metronidazole therapy. J Infect Dis 2006;194:828-36.

Bresson L, Massoni S, Jailloux-Beaurain C, Bissinger MC, Subtil D, Husson MO, Courcol R, Fruchart A. Self-collected vaginal swabs to diagnosis bacterial vaginosis during pregnancy: A pilot study. Gynecol Obstet Fertil 2006;34:701-5.

Briggs AH, Gray AM, Power and sample size calculations for stochastic cost-effectiveness analysis. Med Decis Making 1998;18 suppl:S81 -S92.

Brocklehurst P, Gordon A, Heatley E, Milan SJ. Antibiotics for treating bacterial vaginosis in pregnancy. Cochrane Database Syst Rev. 2013 Jan 31;1:CD000262.

Carey JC, Klebanoff MA, Hauth JC, et al. Metronidazole to prevent preterm delivery in pregnant women with asymptomatic bacterial vaginosis. National Institute of Child Health and Human Development Network of Maternal-Fetal Medicine Units. *N EJM*. 2000;342(8):534–540.

Carey JC, Klebanoff MA. Is a change in the vaginal flora associated with an increased risk of preterm birth? Am J Obstet Gynecol. 2005 Apr;192(4):1341-6.

Cartwright CP, Lembke BD, Ramachandran K, Body BA, Nye MB, Rivers CA, Schwebke JR. J Clin Microbiol. 2012 Jul;50(7):2321-9. Development and validation of a semiquantitative, multitarget PCR assay for diagnosis of bacterial vaginosis.

Cauci S, Driussi S, De Santo D, Penacchioni P, Iannicelli T, Lanzafame P, De Seta F, Quadrifoglio F, de Aloysio D, Guaschino S. Prevalence of bacterial vaginosis and vaginal flora changes in peri- and postmenopausal women. J Clin Microbiol 2002;40:2147-52.

Cauci S, Thorsen P, Schendel DE et al. Determination of immunoglobulin A against Gardenerlla vaginalis hemolysin, sialidase, and prolidase activities in vaginal fluid: implications for adverse pregnancy outcomes. J Clin Microbiol 2003;41:435-8.

CES. Methodological guide for the economic evaluation of health strategies. Paris: Collège des Economistes de la Santé; 2003.

CES: Methodological guide for the implementation of a budget impact analysis. Paris: Collège des Economistes de la Santé; 2008.

Chernesky MA, Hook EW, Martin DH, Lane J, Johnson R, Jordan JA, Fuller D, Willis DE, Fine PM, Janda WM, Schachter J. Women find it easy and prefer to collect their own vaginal swabs to diagnose *Chlamydia trachomatis* or *Neisseria gonorrhoeae* infections. Sex Transm Dis 2005;32:729-33.

Claxton K, Sculpher M, McCabe C, et al. Probabilistic sensitivity analysis for NICE technology assessment: not an optional extra. Health Econ. 2005;14:339–47.

Clerc O, Greub G. Routine use of point-of-care tests: usefulness and application in clinical microbiology. Clin Microbiol Infect. 2010 Aug;16(8):1054-61

Clerc O, Greub G. Clin Microbiol Infect. 2010 Aug;16(8):1054-61; Freifeld AG, Simonsen KA, Booth CS, Zhao X, Whitney SE, Karre T, Iwen PC, Viljoen HJ. A new rapid method for Clostridium difficile DNA extraction and detection in stool: toward point-of-care diagnostic testing. J Mol Diagn. 2012 May-Jun;14(3):274-9. Epub 2012 Mar 5.

CNGOF (French national college of obstetricians and gynecologists): 1999: Recommendations for clinical practice. Infection and pregnancy. www.cngof.fr

Cohen-Bacrie S, Ninove L, Nougairède A, Charrel R, Richet H, Minodier P, Badiaga S, Noël G, La Scola B, de Lamballerie X, Drancourt M, Raoult D. PLoS One. 2011;6:e22403.

Créquat J, Duyme M, Brodaty G. Biometry 2000. Fetal growth charts by the French College of fetal ultrasonography and the Inserm U 155]. Gynecol Obstet Fertil. 2000 Jun;28(6):435-45.

Cristiano L, Rampello S, Noris C, Valota V. Bacterial vaginosis: prevalence in an Italian

population of asymptomatic pregnant women and diagnostic aspects. Eur J Epidemiol 1996;12:383-90.

Culhane JF, Nyirjesy P, McCollum K et al. Variation in vaginal immune parameters and microbial hydrolytic enzymes in bacterial vaginosis positive pregnant women with and without Mobiluncus species. Am J Obstet Gynecol 2006;195:516-21.

De Backer E, Verhelst R, Verstraelen H, Claeys G, Verschraegen G, Temmerman M, Vaneechoutte M. Antibiotic susceptibility of Atopobium vaginae. BMC Infect Dis 2006;6:51.

Department of transport. Highway economics note 1: 2005 valuation of the benefits of prevention of road accidents and casualties; 2007. Available online at: http;//www.dft.gov.uk/pgr/roadsafety/ea/pdfeconnote105.pdf

De Peretti, C., Siani, C. The performance of Fieller's method in problematic cases often occurring in practice Health and System Science 2006 9(1-2), 205-226.

Donders GG. Definition and classification of abnormal vaginal flora. Best Pract Res Clin Obstet Gynaecol 2007;21:355-73.

Donders GG, Van Calsteren K, Bellen G, Reybrouck R, Van den Bosch T, Riphagen I, Van Lierde S. Predictive value for preterm birth of abnormal vaginal flora, bacterial vaginosis and aerobic vaginitis during the first trimester of pregnancy. BJOG. 2009 Sep;116(10):1315-24.

Drummond MF, Sculpher MJ, Torrance GW, O'Brien BJ, Stoddart GL. Methods for the economic evaluation of health care programmes. Third edition ed.: Oxford University press; 2005.

Edejer T, Baltussen R, Adam T, Hutubessy R, Acharya A, Evans D, Murray C. Making choices in Health: WHO guide to cost-effectiveness analysis. Geneva: World Health Organisation; 2003.

Ferris MJ, Masztal A, Aldridge KE, Fortenberry JD, Fidel PL Jr, Martin DH. Association of Atopobium vaginae, a recently described metronidazole resistant anaerobe, with bacterial vaginosis. BMC Infect Dis 2004;4:5.

Flynn CA, Helwig AL, Meurer LN. J Fam Pract. 1999 Nov;48(11):885-92. Bacterial vaginosis in pregnancy and the risk of prematurity: a meta-analysis.

Fredricks DN, Fiedler TL, Marrazzo JM. Molecular identification of bacteria associated with bacterial vaginosis. N Engl J Med 2005;353:1899-911.

Fredricks DN, Fiedler TL, Thomas KK, Mitchell CM, Marrazzo JM. Changes in Vaginal Bacterial Concentrations with Intravaginal Metronidazole Therapy for Bacterial Vaginosis as Assessed by Quantitative PCR. J Clin Microbiol 2009;47:721-726.

Gallo MF, Warner L, Macaluso M, Stone KM, Brill I, Fleenor ME, Hook EW 3rd, Austin HD, Lee FK, Nahmias AJ. Sex Transm Dis 2008;35:679-85.

Gelber SE, Aguilar JL, Lewis KL, Ratner AJ. Functional and physiogenetic characterization of vaginolysin, the human-specific cytolysin from Garnerella vaginalis. J Bacteriol 2008;190:3896-903

Genc MR, Witkin SS, Delaney ML et al. A disproportionate increase in IL-1 beta over IL-1 ra in the cervicovaginal secretions of pregnant women with altered vaginal microflora correlates with preterm birth. Am J Obstet Gynecol 2004;190:1191-7.

Genc MR, Onderdonk AB, Vardhana S et al. Polymorphism in intron 2 of the interleukin-1 receptor antagonist gene, local midtrimester cytokine response to vaginal flora, and

subsequent preterm birth. Am J Obstet Gynecol 2004;191:1324-30.

Genc MR, Vardhana S, Delaney ML et al. TNFA-308G>A polymorphism influences the TNF-alpha response to altered vaginal flora. Eur J Obstet Gynecol Reprod Biol 2007;134:188-30.

Goldenberg RL, Hauth JC, Andrews WW Intrauterine infection and preterm deliveryN Engl J Med. 2000 May 18; 342(20):1500-7

Goldenberg RL, Andrews WW, Faye-Petersen O, Cliver S, Goepfert AR, Hauth JC. The Alabama Preterm Birth Project: placental histology in recurrent spontaneous and indicated preterm birth. Am J Obstet Gynecol. 2006 Sep; 195(3):792-6

Goldenberg RL, Culhane JF, Iams JD, Romero R. Epidemiology and causes of preterm birth. The Lancet. 2008;371(9606):75–84.

Guidelines for the management of sexually transmitted infections. World Health Organization 2005. http://whqlibdoc.who.int/publications/2005/9242546267.pdf

HAS. Antenatal prevention of early neonatal bacterial infection risk. Recommendation 2001. http://www.has-santé.fr

French National Health Authority (HAS). Methodological choice for the economic evaluation at HAS. Methodological Guide October 2011.

Hauth JC, Goldenberg RL, Andrews WW, DuBard MB, Copper RL. Reduced incidence of preterm delivery with metronidazole and erythromycin in women with bacterial vaginosis. N Engl J Med 1995;333:1732-6.

Hay PE, Lamont RF, Taylor-Robinson D, Morgan DJ, Ison C, Pearson J. Abnormal bacterial colonisation of the genital tract and subsequent preterm delivery and late miscarriage. BMJ 1994;308:295-8.

Jenny SL, Hu Y, Overduin P, Meijer A. J Clin Virol. 2010 Oct;49(2):85-9.)

Joesoef M, Schmid G. Bacterial vaginosis. Clin Evid. 2002 Dec;(8):1592-600.

Johansson P. On the definition and age-dependency of the value of a statistical life. J Risk Uncertainty 2002; 25(3):251-63.

Jones BM, Kinghorn GR, Duerden BI. In vitro activity of Azithromycin and Erythromycin against organisms associated with bacterial vaginosis and chancroid. Eur J Clin Microbiol Infect Dis. 1988 Aug;7(4):551-3..

Kalinka J, Sobala W, Wasiela M, Brzezinska-Blaszczyk E. Decreased proinflammatory cytokines in cervicovaginal fluid, as measured in medgestation, are associated with preterm delivery. Am J Reprod Immunol 2005;54:70-6.

Kaul R, Nagelkerke NJ, Kimani J, Ngugi E, Bwayo JJ, Macdonald KS, Rebbaprgada A, Fonck K, Temmerman M, Ronald AR, Moses S; Kibera HIV Study Group. Prevalent herpes simplex virus type 2 infection is associated with altered vaginal flora and an increased susceptibility to multiple sexually transmitted infections. J Infect Dis 2007;196:1692-7.

Kiss H, Petricevic L, Husslein P. Prospective randomized controlled trial of an infection screening programme to reduce the rate of preterm delivery. BMJ. 2004 Aug 14;329(7462):371.

Klebanoff MA, Schwebke JR, Zhang J, Nansel TR, Yu KF, Andrews WW. Vulvovaginal symptoms in women with bacterial vaginosis. Obstet Gynecol 2004;104: 267-72.

Knox J, Tabrizi SN, Miller P, Petoumenos K, Law M, Chen S, Garland SM. Evaluation of self-collected samples in contrast to practitioner-collected samples for detection of *Chlamydia trachomatis*, *Neisseria gonorrhoeae*, and *Trichomonas vaginalis* by polymerase chain reaction among women living in remote areas. Sex Transm Dis 2002;29:647-54.

Koopmanschap MA, Rutten FF, van Ineveld BM, van Roijen L. The friction cost method for measuring indirect costs of disease. J Health Econ. 1995 Jun;14(2):171-89.

Rautava L, Andersson S, Gissler M, Hallman M, Häkkinen U, Peltola M, Tammela O, Lehtonen L. Hospital costs and quality of life during 4 years after very preterm birth. Arch Pediatr Adolesc Med. 2010 Jul;164(7):657-63. doi: 10.1001/archpediatrics.2010.99.

Krauss-Silva L, Moreira ME, Alves MB, Braga A, Camacho KG, Batista MR, Almada-Horta A, Rebello MR, Guerra F. A randomised controlled trial of probiotics for the prevention of spontaneous preterm delivery associated with bacterial vaginosis: preliminary results. Trials. 2011 Nov 8;12:239.

Lamont RF, Taylor-Robinson D. Review of the accuracy of various diagnostic tests for bacterial vaginosis to predict preterm birth). BJOG. 2005 Feb;112(2):259-60

Lamont RF, Nhan-Chang CL, Sobel JD, Workowski K, Conde-Agudelo A, Romero R. Treatment of abnormal vaginal flora in early pregnancy with clindamycin for the prevention of spontaneous preterm birth: a systematic review and meta analysis. Am J Obstet Gynecol. 2011 Sep;205(3):177-90.

Larsson PG, Platz-Christensen JJ, Thejls H, Forsum U, Påhlson C. Incidence of pelvic inflammatory disease after first-trimester legal abortion in women with bacterial vaginosis after treatment with metronidazole: a double-blind, randomized study. Am J Obstet Gynecol 1992;166:100-3.

Larsson PG, Carlsson B, Fahraeus L, Jakobsson T, Forsum U. Diagnosis of bacterial vaginosis: need for validation of microscopic image area used for scoring bacterial morphotypes. Sex Transm Infect 2004;80:63-7.

Larsson PG, Fahraeus L, Carlsson B, Jakobsson T, Forsum U. Predisposing factors for bacterial vaginosis, treatment efficacy and pregnancy outcome among term deliveries; results from a preterm delivery study. BMC Womens Health 2007;7:20.

Leitich H, Bodner-Adler B, Brunbauer M et al. Bacterial vaginosis as a risk factor for preterm delivery: a meta-analysis. Am J Obstet Gynecol. 2003;189:139-47.

Leitich H, Kiss H. Asymptomatic bacterial vaginosis and intermediate flora as risk factors for adverse pregnancy outcome. Best Pract Res Clin Obstet Gynaecol 2007;21:375-90.

Libman MD, Kramer M, Platt R, Montreal Prematurity Study Group. Comparison of Gram and Kopeloff stains in the diagnosis of bacterial vaginosis in pregnancy. Diagn Microbiol Infect Dis 2006;54:197-201.

Lin L, Song J, Kimber N, Shott S, Tangora J, Aroutcheva A, Mazees MB, Wells A, Cohen A, Faro S. The role of bacterial vaginosis in infection after major gynecologic surgery. Infect Dis Obstet Gynecol 1999;7:169-74.

Lucey T. Costing. United Kingdom: Thompson Learning; 2002.

McDonald HM, O'Loughlin JA, Vigneswaran R, Jolley PT, Harvey JA, Bof A, et al. Impact of metronidazole therapy on preterm birth in women with bacterial vaginosis flora (Gardnerella vaginalis): a randomised, placebo controlled trial. Br J Obstet Gynaecol 1997;

104:1391–7.

McDonald HM, Brocklehurst P, Gordon A. Antibiotics for treating bacterial vaginosis in pregnancy. Cochrane Database of Systematic Reviews 2007, Issue 1. Art. No.: CD000262.

Macones GA, Parry S, Elkousy M et al. A polymorphism in the promoter region of TNF and bacterial vaginosis: preliminary evidence of gene-environment interation in the etiology of spontaneous preterm birth. Am J Obstet Gynecol 2004;190:1504-8.

Mangham L., Petrou S., Doyle, L., Draper, E. Marlow, N. The cost of preterm birth throughout childhood in England and Wales Pediatrics 2009 vol 123 (2).

Mangot-Bertrand J, Fenollar F, Bretelle F, Gamerre M, Raoult D, Courbiere B. Molecular diagnosis of bacterial vaginosis: impact on IVF outcome. Eur J Clin Microbiol Infect Dis. 2013 Apr;32(4):535-4.

Marconi C, Cruciani F, Vitali B, Donders GG. J Correlation of Atopobium vaginae Amount With Bacterial Vaginosis Markers. Low Genit Tract Dis. 2012 Apr;16(2):127-32.

Marrazzo JM, Koutsky LA, Eschenbach DA, Agnew K, Stine K, Hillier SL. Characterization of vaginal flora and bacterial vaginosis in women who have sex with women. J Infect Dis 2002;185:1307-13.

Martin HL, Richardson BA, Nyange PM, Lavreys L, Hillier SL, Chohan B, Mandaliya K, Ndinya-Achola JO, Bwayo J, Kreiss J. Vaginal lactobacilli, microbial flora, and risk of human immunodeficiency virus type 1 and sexually transmitted disease acquisition. J Infect Dis 1999;180:1863-8.

Mediannikov O, Fenollar F, Bassene H, Tall A, Sokhna C, Trape JF, Raoult D. Description of "yaaf", the vesicular fever caused by acute Rickettsia felis infection in Senegal. J Infect. 2012 Oct 13.

Menard JP, Fenollar F, Henry M, Bretelle F, Raoult D. Molecular quantification of *Gardnerella vaginalis* and *Atopobium vaginae* loads to predict bacterial vaginosis. Clin Infect Dis 2008; 47:33-43.

Menard JP, Bretelle F. Cervicovaginal infections during pregnancy (recommendations). EMC Obstetrics 5-047-G30.

Menard JP (A), Mazouni C, Fenollar F, Raoult D, Boubli L, Bretelle F. Diagnostic accuracy of quantitative real-time PCR assay versus clinical and Gram stain identification of bacterial vaginosis. Eur J Clin Microbiol Infect Dis. 2010 Dec;29(12):1547-52.

Menard JP (B), Mazouni C, Salem-Cherif I, Fenollar F, Raoult D, Boubli L, Gamerre M, Bretelle F. High vaginal concentrations of Atopobium vaginae and Gardnerella vaginalis in women undergoing preterm labor. Obstet Gynecol. 2010 Jan;115(1):134-40.

Menard JP. Antibacterial treatment of bacterial vaginosis: current and emerging therapies. Int J Womens Health. 2011;3:295-305.

Menard JP (A), Fenollar F, Raoult D, Boubli L, Bretelle F. Self-collected vaginal swabs for the quantitative real-time polymerase chain reaction assay of *Atopobium vaginae* and *Gardnerella vaginalis* and the diagnosis of bacterial vaginosis. Eur J Clin Microbiol Infect Dis. 2012;31:513-8.

Menard JP (B) , Bretelle F. Bacterial vaginosis and preterm delivery]. Gynecol Obstet Fertil. 2012 Jan;40(1):48-54.

Menard JP, Bretelle F (C). How can the treatment of bacterial vaginosis be improved to

reduce the risk of preterm delivery? Womens Health (Lond Engl). 2012 Sep;8(5):491-3.

Merritt AJ, Levy A, Vietheer P, Bradbury R, Scholler A, Chidlow G, Smith DW. Deployable laboratory response to influenza pandemic; PCR assay field trials and comparison with reference methods. PLoS One. 2011;6(10):e25526. Epub 2011 Oct 12.

Mitchell CM, Hitti JE, Agnew KJ, Fredricks DN. Comparison of oral and vaginal metronidazole for treatment of bacterial vaginosis in pregnancy: impact on fastidious bacteria. BMC Infect Dis 2009;9:89.

Morales WJ, Schorr S, Albritton J. Effect of metronidazole in patients with preterm birth in preceding pregnancy and bacterial vaginosis: a placebo-controlled, double-blind study. Am J Obstet Gynecol. 1994 Aug;171(2):345-7

Myhre R, Brantsæter AL, Myking S et al. Intake of probiotic food and risk of spontaneous preterm delivery. Am. J. Clin. Nutr. 93(1), 151–157 (2011).

Nelson DB, Bellamy S, Gray TS, Nachamkin I. Self-collected versus provider-collected vaginal swabs for the diagnosis of bacterial vaginosis: an assessment of validity and reliability. J Clin Epidemio 2003:862-866.

Ness RB, Kip KE, Hillier SL, Soper DE, Stamm CA, Sweet RL, Rice P, Richter HE.A cluster analysis of bacterial vaginosis-associated microflora and pelvic inflammatory disease. Am J Epidemiol 2005;162:585-90.

Nougairede A, Ninove L, Zandotti C, de Lamballerie X, Gazin C, Drancourt M, La Scola B, Raoult D, Charrel RN. Point of care strategy for rapid diagnosis of novel A/H1N1 influenza virus. PLoS One. 2010 Feb 17;5(2):e9215).

Nugent RP, Krohn MA, Hillier SL. Reliability of diagnosing bacterial vaginosis is improved by a standardized method of gram stain interpretation. J Clin Microbiol 1991;29:297-301.

Núñez JT, Gómez G. Low-dose secnidazole in the treatment of bacterial vaginosis. Int J Gynaecol Obstet 2005;88:281-5.

Odendaal HJ, Popov I, Schoeman J, Smith M, Grové D. Preterm labour--is bacterial vaginosis involved? S Afr Med J. 2002 Mar;92(3):231-4.

Oduyebo OO, Anorlu RI, Ogunsola FT. The effects of antimicrobial therapy on bacterial vaginosis in non-pregnant women. Cochrane Database Syst Rev. 2009:CD006055.

Ogilvie GS, Patrick DM, Schulzer M, Sellors JW, Petric M, Chambers K, White R, FitzGerald JM. Diagnostic accuracy of self collected vaginal specimens for human papillomavirus compared to clinician collected human papillomavirus specimens: a meta-analysis. Sex Transm Infect 2005;81:207-12.

Okun N, Gronau KA, Hannah ME. Antibiotics for bacterial vaginosis or Trichomonas vaginalis in pregnancy: a systematic review. Obstet Gynecol. 2005 Apr;105(4):857-68.

Othman M, Neilson JP, Alfirevic Z.Probiotics for preventing preterm labour. Cochrane Database Syst. Rev. 1, CD005941 (2007).

Petignat P, Hankins C, Walmsley S, Money D, Provencher D, Pourreaux K, Kornegay J, Rouah F, Coutlée F; Canadian Women's HIV Study Group. Self-sampling is associated with increased detection of human papillomavirus DNA in the genital tract of HIV-seropositive women. Clin Infect Dis 2005;41:527-34.

Petrou S., Khan K. Economic costs associated with moderate and late preterm birth: primary and secondary evidence Seminars in fetal and neonatal medicine 2012: 17:170-178.

Schwebke JR, Desmond R. A randomized trial of metronidazole in asymptomatic bacterial vaginosis to prevent the acquisition of sexually transmitted diseases. Am J Obstet Gynecol 2007;196:517.e1-6.

Senok AC, Verstraelen H, Temmerman M, Botta GA. Probiotics for the treatment of bacterial vaginosis. Cochrane Database Syst Rev 2009:CD006289.

Sewankambo N, Gray RH, Wawer MJ, Paxton L, McNaim D, Wabwire-Mangen F, Serwadda D, Li C, Kiwanuka N, Hillier SL, Rabe L, Gaydos CA, Quinn TC, Konde-Lule J. HIV-1 infection associated with abnormal vaginal flora morphology and bacterial vaginosis. Lancet 1997;350:546-50.

Sexually Transmitted Diseases Treatment Guidelines 2006. US Centers for Disease Control and Prevention Website.

Siani, C., De Peretti, C. How to make decision with the incremental Cost-Effectiveness Ratio under uncertainty? Health and System Science 2006 9(1-2), 111-145.

Simhan HN, Caritis SN, Krohn MA et al. Decreased cervical proinflammatory cytokines permit subsequent upper genital infection during pregnancy. Am J Obstet Gynecol 2003;189:560-7.

Strauss RA, Eucker B, Savitz DA, Thorp JM Jr. Diagnosis of bacterial vaginosis from self-obtained vaginal swabs. Infect Dis Obstet Gynecol 2005;13:31-5.

Svare JA, Schmidt H, Hansen BB, Lose G. Bacterial vaginosis in a cohort of Danish pregnant women: prevalence and relationship with preterm delivery, low birthweight and perinatal infections. BJOG 2006;113:1419-25.

Theunissen NC, Veen S, Fekkes M, Koopman HM, Zwinderman KA, Brugman E, Wit JM. Quality of life in preschool children born preterm. Dev Med Child Neurol. 2001;43(7):460–465.

Thorsen P, Jensen IP, Jeune B et al. Few microorganisms associated with bacterial vaginosis may constitute the pathologic core: a population_based microbiologic study among 3596 pregnant women. Am J Obstet Gynecol 1998;178:580-7.

Thorsen P, Vogel I, Molsted K et al. Risk factors for bacterial vaginosis in pregnancy: a population-based study on Danish women. Acta Obstet Gynecol Scand 2006; 85:906-11.

Trabert B, Misra DP. Risk factors for bacterial vaginosis during pregnancy among African American women. Am J Obstet Gynecol 2007;197:477.e1-8.

Ugwumadu A, Reid F, Hay P, Manyonda I. Natural history of bacterial vaginosis and intermediate flora in pregnancy and effect of oral clindamycin. Obstet Gynecol 2004;104:114-9.

Ugwumadu A. Role of antibiotic therapy for bacterial vaginosis and intermediate flora in pregnancy. Best Pract Res Clin Obstet Gynaecol 2007;21:391-402.

Van Roijen L, Essink-Bot M, Koopmanschap MA., Bonsel, G., Rutten F. Labor and health status in economic evaluation of health care. Int J Technol Assess Health Care. 1996; 12(3): 405-15.

Vogel I, Thorsen P, Jeune B et al. Acquisition and elimination of bacterial vaginosis during pregnancy: a Danish population-based study. Infect Dis Obstet Gynecol 2006;94646.

Waller J, McCaffery K, Forrest S, Szarewski A, Cadman L, Austin J, Wardle J. Acceptability of unsupervised HPV self-sampling using written instructions. J Med Screen 2006;13:208-13.

Ware JE, Kosinski M, Turner-Bowker DM, Gandeck B. User's manual for the SF-12v2™ Health Survey: (with a supplement documenting SF-12® Health Survey). Lincoln: QualityMetric; 2002.

Wiesenfeld HC, Hillier SL, Krohn MA, Landers DV, Sweet RL. Bacterial vaginosis is a strong predictor of Neisseria gonorrhoeae and Chlamydia trachomatis infection. Clin Infect Dis 2003;36:663-8.

Zwicker JG, Harris SR. Quality of life of formerly preterm and very low birth weight infants from preschool age to adulthood: a systematic review. Pediatrics. 2008;121(2):e366–e376. doi: 10.1542/peds.2007-0169.

2014-001559-22 Version 8 dated September 6, 2017
