# Supplementary material for: Effectiveness and Costs of Molecular Screening and Treatment for Bacterial Vaginosis to Prevent Preterm Birth: The AuTop Randomized Clinical Trial
Source: JAMA Pediatr. 2023 Jul 17;177(9):894–902. doi: 10.1001/jamapediatrics.2023.2250 (PMC10352927; doi:10.1001/jamapediatrics.2023.2250)
Supplement: Supplement 2. — Statistical Analysis Plan [file jamapediatr-e232250-s002.pdf]

The rationale page that appears between the title page and the Table Of Content (TOC) is for amended SAPs only.

Date	Version	History of main changes in the model document	Impacted Section	Source
15-01-2015	1	NA	NA	NA
12-06-2017	2	ICER per preterm birth avoided before 24, 26, 28, and 32 WG as secondary outcomes deleted	2.4	

STATISTICAL ANALYSIS PLAN For INTERVENTIONAL STUDIES

**TITLE (English):**	**AuTop trial : Screen-and-treat program by Point-of-care of *Atopobium vaginae* and *Gardnerella vaginalis* in preventing preterm birth**
**Title (French):**	**Impact médico-économique du dépistage d'atopobium vaginae et de gardnerella vaginalis en biologie moléculaire par "point of care" lors de la grossesse**
**PROTOCOL NUMBER:**	2 (date 23-05-2014)
**VERSION NUMBER:**	2.0
**STUDIED MEDICINAL PRODUCT:**	None
**PLAN PREPARED BY:**	Anderson Loundou & Sandrine Loubière<br>Statistician and health economist respectively<br>Service épidémiologie et économie de la santé, APHM
**DATE FINAL:**	12-06-2017
**STUDY INITIATOR:**	Pr Florence Bretelle<br>Department of Gynaecology and Obstetrics, Hospital Nord, APHM

TABLE OF CONTENTS

1. BACKGROUND AND STUDY OBJECTIVES 4
   1.1 Background 4
   1.2 Study objectives 4
2. STUDY DESIGN 4
   2.1 Description of Study Design 5
   2.2 Data Sources 5
   2.3 Primary Outcome MeasureS 5
      2.3.1 2.3.1 Primary clinical outcome 6
      2.3.2 Primary economic outcome 6
   2.4 Secondary Outcome Measures 6
   2.5 Other Outcome Measures 7
   2.6 Determination of Sample Size 7
3. STATISTICAL METHODS 8
   3.1 Populations TARGETED 8
   3.2 Effectiveness Analysis 8
      3.2.1 Primary Clinical Outcome 8
      3.2.2 Primary Economic Outcome 9
      3.2.3 Secondary Outcome Measures 9
   3.3 Subgroup analyses 10
   3.4 Safety Analyses 10
   3.5 Sensitivity Analyses 10
   3.6 Handling of Missing Data 10
   3.7 Interim, Final Analyses and Timing of Analyses 10
4. REFERENCES 11

1. BACKGROUND AND STUDY OBJECTIVES

1.1 BACKGROUND

International recommendations in favor of screening for vaginal infection in pregnancy are based on heterogeneous criteria. In most developed countries, the diagnosis of bacterial vaginosis is only recommended for women with high-risk of preterm birth. Nugent score is currently used, but molecular quantification tools have been reported with a high sensitivity and specificity. Their value for reducing preterm birth rates and related complications remains unexplored. This trial was designed to assess the cost-effectiveness of a systematic screen-and-treat program based on a Point-of-care technique for rapid molecular diagnosis, immediately followed by an appropriate antibiotic treatment, to detect the presence of abnormal vaginal flora (more specifically *Atopobium vaginae* and *Gardnerella vaginalis*) before 20 weeks of gestation in pregnant women in France. We hypothesized that this program would translate into significant reductions in both rate of preterm births and medical costs associated with preterm birth.

1.2 STUDY OBJECTIVES

To assess the cost-effectiveness of the innovative screening for *A. vaginae* and *G. vaginalis* portage using a molecular quantification method by point-of-care with an appropriate treatment for positive cases, compared to a usual care strategy in pregnant women at less than 20 weeks of gestation.

2. STUDY DESIGN

The study was designed using the recommendations of the Consolidated Standards of Reporting Trials (CONSORT) statement and according to the guidelines of cost-effectiveness studies of the French Health Authority [Haute Autorité en Santé, Choix

méthodologiques pour l'évaluation économique à la HAS http://www.has-sante.fr/portail/jcms/c_1120711/choix-methodologiques-pour-l-evaluation-economique-a-la-has].

2.1 DESCRIPTION OF STUDY DESIGN

A multicenter, open-label randomized controlled, two-parallel group study was designed in which pregnant women who attend prenatal care consultations before 20 weeks' gestation at French obstetrics and gynecology centers are randomized between two management strategies: systematic vaginosis screen-and-treat strategy (experimental group) and usual care management (control group).

2.2 DATA SOURCES

All data are recorded from an electronic case report form (eCRF) specifically elaborated for the study (eCRF CleanWEB, Telemedicine Technologies S.A.S., www.tentelemed.com, 2015) and recorded at four specific study's times as follows: randomization (T0), baseline assessment (T1), delivery (T2), and at 6 months after delivery (T3). All assessments are based either on medical files (pregnancy and delivery characteristics, obstetrical and neonatal outcomes), face-to-face questionnaires (smoking and alcohol habits, personal hygiene, pregnancies history or symptoms, concomitant treatments such as treatment with pessary or progesterone), phone calls (to collect data on vaginal symptoms or potential side-effects of antibiotic) or self-report (health outcomes and health service use during the pregnancy and 6 months following the initial hospitalization).

2.3 PRIMARY OUTCOME MEASURES

2.3.1 2.3.1 Primary clinical outcome

The rate of preterm birth avoided of the screen and treat strategy compared to the usual care. The effectiveness criterion has been discussed and consensually approved by all the study's main partners (gynecologist coordinator and co-coordinators, biologists, health economist, methodologist).

2.3.2 Primary economic outcome

The medico-economic endpoint is the incremental cost-effectiveness ratio (ICER), expressed as the extra cost per additional preterm birth avoided of the screen and treat strategy compared to the usual care.

2.4 SECONDARY OUTCOME MEASURES

- Obstetrical outcomes: rates of preterm birth before 24, 26, 28, 32, and 37 weeks of gestation, spontaneous abortion, premature rupture of membranes, severe intrauterine growth restriction, preterm labor, duration of the woman's hospitalization;
- Neonatal outcomes: neonatal mortality, neonatal morbidity (respiratory distress syndrome, bronchopulmonary dysplasia, necrotizing enterocolitis, periventricular leukomalacia), transfer to a neonatal intensive care unit (duration), mechanical ventilation (duration), congenital anomalies, duration of the newborn's hospitalization;
- Treatment effectiveness: rate of recurrence (defined as a positive control vaginal swab using qPCR after the negativation of a precedent control vaginal swab), rate of treatment failure (defined as *A. vaginae* $\geq 10^8$copies/mL and/or a *G. vaginalis* load $\geq 10^9$copies/mL from a control vaginal swab) and side-effects associated with treatment;

- Health care utilization: all the mother's use of health care during the whole study period (e.g., gynecologists and general practitioner consultations, hospital admission, clinical examinations and medications), as well as health care for the newborn (including neonatal care, re-hospitalization, medications, planned and non-planned consultations with pediatric practitioner or other specialists).
-

2.5 OTHER OUTCOME MEASURES

NA

2.6 DETERMINATION OF SAMPLE SIZE

The sample size was calculated from the expected differential ICER per preterm birth avoided between the 2 groups. In accordance with Briggs [1], the following hypothesis is stated: with an expected incremental rate of preterm birth of 1.3% (4.3% in the control group [2] and 3.0% in the experimental group), an expected incremental cost of 230 euros (including cost of initial and following point-of-care tests and cost of treatments for 10% of the women [3]), and an estimated threshold at 22,500 euros, corresponding to the avoided cost of a preterm birth before 37 weeks [4]. With an 80% statistical power and a threshold for statistical significance set at a p-value of 0.05, and assuming that a potential 20% of patients will be lost to follow-up, these calculations showed that 6,800 patients are needed (3,400 per group). Considering the potential of inclusion of each participating center, the inclusion duration will be planned over a 12-month period. The maximal period of participation for the included women is 12 months.

3. STATISTICAL METHODS

3.1 POPULATIONS TARGETED

The main inclusion criteria are: women must have less than 20 weeks of gestation, with singleton pregnancy, they must be symptomatic or non-symptomatic as regards the diagnosis of BV, and they must not have high-risk factors of preterm birth.

The main exclusion criteria are high-risk factors of preterm birth (such as diabetes, hypertension, fetal/uterine malformation, multiple pregnancy, or a history of preterm birth).

3.2 EFFECTIVENESS ANALYSIS

3.2.1 Primary Clinical Outcome

The first comparison of the 2 groups based on preterm birth rate before 37 weeks' gestation will be carried out using a chi-square test.

To assess whether the effect of treatment on the preterm birth rate varied with prognostic covariates, a multivariate analysis will be performed using logistic regression models. The variable to be explained will be represented by the 37 weeks' preterm birth yes/no variable; the selection of the explanatory variables will be based, on the one hand, on the univariate approach which will identify variables for which the *P* value is lower than or equal to 0.20 (the group variable will be automatically selected), and, on the other hand, on the prior identification of variables potentially associated with preterm birth (tobacco use, age, BMI, Caucasian, vaginal hygiene, previous miscarriage, induced pregnancy). The results will be presented in the form of odds ratios and their confidence intervals. Statistical significance will be defined as $P < 0.05$.

Statistical analysis will be performed with R.

3.2.2 Primary Economic Outcome

Thus, the ICER provides information on the potential acceptability of the intervention for decision-makers. The costs perspective taken in our economic analysis is that of the healthcare payer. The time horizon starts from the first prenatal consultation before the 20 weeks of gestation and ended at the discharge of neonates or death. The healthcare costs included are those that are likely to differ across the intervention and control groups. In our study these costs are those associated with: screening using the point-of-care procedure (quantitative molecular analysis), control vaginal swabs for positive women, antibiotic treatments, antenatal hospital admissions, physicians' consultations, management of complications, as well as neonatal costs for full term infants and preterm infants. Unit costs for health service use will be estimated using data from the French National Hospital Database (Programme de médicalisation des systèmes d'information, PMSI) and National tariffs. Treatment costs will be obtained from the French register of pharmaceutical specialties, an online database of information on healthcare products. All resources will be valued in 2020 euros, and there is no requirement to apply discounting.

3.2.3 Secondary Outcome Measures

Continuous variables will be expressed as mean ± standard deviation (SD), and categorical variables reported as counts and percentages. Mean values will be compared with the Student t test or the Mann–Whitney U test, and percentages compared with the $\chi 2$ test or Fisher exact test, as appropriate.

The thresholds will be chosen from the analysis of diagnostic effectiveness (accuracy) and expressed as the proportion of correctly classified subjects among all subjects. A high

vaginal load of *A. vaginae* (DNA level $\geq 10^8$ copies/mL) identifies a population at high risk of preterm birth. According to previous works [5-7], BV will be defined by an *A. vaginae* load $\geq 10^8$copies/mL and/or a *G. vaginalis* load $\geq 10^9$copies /mL.

3.3 SUBGROUP ANALYSES

Unadjusted and adjusted subgroup analyses for the primary outcome will be performed using CRR. Data for subgroups will be presented as a Forest plot (see Figure 3 - adjusted OR, 95%CI). The subgroups are defined according to the following variables: nulliparous vs. multiparous women, women age (mean); tobacco smoker vs. nonsmoker.

3.4 SAFETY ANALYSES

Please see Annex 1 for details Safety assessment

3.5 SENSITIVITY ANALYSES

Probabilistic sensitivity analyses, using the non-parametric bootstrap method, will be carried out to generate mean expected ICERs and to determine whether uncertainty or variation in the data used affect the ICER. In addition, cost-effectiveness acceptability curves will be constructed to represent decision uncertainty surrounding cost-effectiveness estimates.

3.6 HANDLING OF MISSING DATA

Missing data will be handled where possible using multiple imputations or other method according to the type of missing data.

3.7 INTERIM, FINAL ANALYSES AND TIMING OF ANALYSES

No interim analysis scheduled.

4. REFERENCES

1. Briggs AH, Gray AM: Power and sample size calculations for stochastic cost-effectiveness analysis. Med Decis Making 1998, 18(2 Suppl):S81-92.

2. Kiss H, Petricevic L, Husslein P: Prospective randomised controlled trial of an infection screening programme to reduce the rate of preterm delivery. BMJ 2004, 329(7462):371.

3. Goffinet F, Maillard F, Mihoubi N, Kayem G, Papiernik E, Cabrol D, Paul G: Bacterial vaginosis: prevalence and predictive value for premature delivery and neonatal infection in women with preterm labour and intact membranes. Eur J Obstet Gynecol Reprod Biol 2003, 108(2):146-151.

4. Petrou S, Khan K: Economic costs associated with moderate and late preterm birth: primary and secondary evidence. Semin Fetal Neonatal Med 2012, 17(3):170-178.

5. Menard JP, Fenollar F, Henry M, Bretelle F, Raoult D: Molecular quantification of Gardnerella vaginalis and Atopobium vaginae loads to predict bacterial vaginosis. Clin Infect Dis 2008, 47(1):33-43.

6. Menard JP, Mazouni C, Salem-Cherif I, Fenollar F, Raoult D, Boubli L, Gamerre M, Bretelle F: High vaginal concentrations of Atopobium vaginae and Gardnerella vaginalis in women undergoing preterm labor. Obstet Gynecol 2010, 115(1):134-140.

7. Bretelle F, Rozenberg P, Pascal A, Favre R, Bohec C, Loundou A, Senat MV, Aissi G, Lesavre N, Brunet J et al: High Atopobium vaginae and Gardnerella vaginalis Vaginal Loads Are Associated With Preterm Birth. Clin Infect Dis 2015, 60(6):860-867.

Appendix 1 SAFETY ASSESSMENT

1. RISKS AND RESTRICTIONS ADDED BY THE STUDY

The diagnostic tests and treatment used for the study is already used in France and all over the world. Their safety are already proven.

Recording and reporting adverse events

1.1 Definitions

According to Article R1123-46 of the French Public Health Code:

- **Adverse event**

Any untoward medical occurrence in a trial subject, which does not necessarily have a causal relationship with the clinical trial or with the investigational product.

- **Adverse reaction to an investigational medicinal product**

Any adverse event occurred in a trial subject, which has a causal relationship with the clinical trial or with the investigational medicinal product

- **Serious adverse event or reaction**

Any adverse event or reaction that at any dose of medication, results in death, threatens the life of the research subject, requires hospitalisation or prolongs hospitalisation, causes a severe or long-term disability or handicap, or results in a congenital abnormality or deformity.

- **Unexpected adverse reaction to an investigational medicinal product**

Any adverse reaction to the product, whose nature, severity, frequency or outcome is inconsistent with the safety information described in the Reference Safety Information (summary of product characteristics, or the investigator's brochure if the product is not authorised).

<u>According to Article R.1123-46 of the Code de la Santé Publique and the guidelines for clinical trial sponsors (ANSM):</u>

- **Emerging safety issue**

Any new safety information that may lead to a reassessment of the risk/benefit ratio of the trial or the investigational medicinal product, modifications in the investigational medicinal product use, the conduct of the clinical trial, or the clinical trial documents, or a suspension, interruption or modification of the protocol of the clinical trial or other similar trials.

For the clinical trials involving the first administration or use of an investigational medicinal product in healthy volunteers, any serious adverse reaction.

Examples:

a) any clinically significant increase in the frequency of an expected serious adverse reaction

b) suspected unexpected serious adverse reactions in patients who have terminated their participation in the clinical trial that are notified by the investigator to the sponsor together with follow-up reports

c) any new safety issue relating to the conduct of the clinical trial or the development of the investigational medicinal product, that may impact the safety of the trial subjects.

Examples:

- a serious adverse event likely to be related to the interventions and the trial's diagnostic procedures and which may impact the conduct of the clinical trial,
- a significant risk on the trial subjects such as ineffectiveness of the investigational medicinal product in treating a life-threatening illness under investigation,
- significant safety results from a recently completed non-clinical study (such as a carcinogenicity study),

- the premature termination, or temporary suspension, of a trial conducted on the same investigational medicinal product in another country, for safety reasons,
- an unexpected serious adverse reaction associated with a non-experimental medication required for the conduct of the clinical trial, (e.g. challenge agents, rescue treatment)
d) any suspected unexpected serious adverse reaction (SUSAR) reported to the sponsor by another sponsor of a trial carried out in a different country but relating to the same medication.

1.2 The role of the investigator

The investigator must **assess the seriousness criteria of each adverse event** and record all serious and non-serious adverse events in the case report form (eCRF).
The investigator must **document** serious adverse events **as thoroughly as possible** and provide a definitive medical diagnosis, if possible.
The investigator must assess the severity of the adverse events by using:
Common Terminology Criteria for Adverse Events [National Cancer Institute]

The investigator must assess the **causal relationship** between the serious adverse events and the investigational medicinal products.
The method used by the investigator is based on the WHO Uppsala Monitoring Centre method and uses the following causality terms:

- Certain
- Probable/likely
- Possible
- Unlikely (not ruled out).

These terms are defined as follows (extracted from the WHO-UMC causality categories, version dated 17/04/2012).

Table N°5: WHO-UMC causality categories (extract)

Causality term	Assessment criteria*
**Certain**	· Event or laboratory test abnormality, with plausible time relationship to drug intake **<br>· Cannot be explained by disease or other drugs<br>· Response to withdrawal plausible (pharmacologically, pathologically)<br>· Event definitive pharmacologically or phenomenologically (i.e. an objective and specific medical disorder or a recognized pharmacological phenomenon)<br>· Rechallenge satisfactory, if necessary
**Probable / Likely**	· Event or laboratory test abnormality, with reasonable time relationship to drug intake**<br>· Unlikely to be attributed to disease or other drugs<br>· Response to withdrawal clinically reasonable<br>· Rechallenge not required
**Possible**	

	· Event or laboratory test abnormality, with reasonable time relationship to drug intake **<br>· Could also be explained by disease or other drugs<br>· Information on drug withdrawal may be lacking or unclear
**Unlikely**	· Event or laboratory test abnormality, with a time to drug intake **<br>· that makes a relationship improbable (but not impossible)<br>· Disease or other drugs provide plausible explanations

*All points should be reasonably complied with
** Or study procedures

1.2.1 Serious adverse events that require a notification without delay by the investigator to the sponsor

As per article R.1123-49 of the French Public Health Code (CSP), the investigator must notify the sponsor **without delay on the day when the investigator becomes aware** of any serious adverse event which occurs during a trial as described in Article L.1121-1(1) CSP, except those which are listed in the protocol (see section 10.1.2.2) and, if applicable, in the investigator's brochure as not requiring a notification without delay. These latter should be notified by the investigator to the sponsor in an appropriate delay taking into consideration the specific features of the trial, the serious adverse events and the modalities specified in the protocol or the investigator's brochure.
A serious adverse event is any untoward medical occurrence that:
1- results in death
2- is life-threatening
3- requires inpatient hospitalisation or prolongation of existing hospitalisation
4- results in persistent or significant disability/incapacity
5- is a congenital anomaly/birth defect

Then, any SAE leading to death (fetal or neonatal) will be notified to the sponsor by the investigator without delay.

1.2.2 Specific features of the protocol: Serious adverse events that do not require the investigator to notify the sponsor without delay
These serious adverse events are simply recorded in the case report form.

Any premature birth will not be notified without delay by the investigator to the sponsor.
As intra-uterine infection is expected in this context, it will not to be notified without delay by the investigator to the sponsor.
The initial systematic hospitalization for close monitoring will not to be notified without delay by the investigator to the sponsor.
Spontaneous labor, labor induction or cesarean section will not to be notified without delay by the investigator to the sponsor.

Immediate infections in the newborn are difficult to be confirmed and cannot be notified without delay by the investigator to the sponsor.
A CRF extraction of these conditions (premature birth, systematic hospitalization, spontaneous labor, labor induction or cesarean section, early- and late-onset sepsis will be realized every 6 months by Clinical Research Unit and sent by email to Safety Department.
Any neonatal death will be notified without delay by the investigator to the sponsor.
If there is any imbalance between the randomization groups or the mortality rate is higher than expected affecting the safety of trial subjects and which requires the sponsor to take urgent safety measures, the ANSM will be informed about the emerging safety issue without delay.

1.2.4 Procedures and deadlines for notifying the sponsor
The investigator should initially complete a SAE reporting form (contained in the case report form). This report must be signed by the investigator.
The investigator must complete every section of the SAE form so that the sponsor can carry out the appropriate assessment.
The initial report sent to the sponsor must be rapidly followed up by one or more additional written reports describing the course of the event and any complementary information.
Whenever possible, the investigator will provide the sponsor with any documents that may be useful for medical assessment of the case (medical reports, laboratory test results, results of additional exams, etc.). These documents must be anonymized. In addition, the investigator must state the study acronym and the number and initials of the study participant on each paper.
Any adverse event will be monitored until fully resolved (stabilisation at a level considered acceptable by the investigator, or return to the previous state) even if the subject has terminated his participation in the trial.
The initial report, the SAE follow-up reports and all other documents must be sent to the sponsor's safety Department by **mail**, (eig-vigilance.drc@aphp.fr). It will be possible to transmit the EIG to the sponsor's safety Department by fax**, only in case of unsuccessfully attempt to send by mail (to avoid duplication)**.
For trials which use e-CRF
- the investigator completes the SAE report form in the e-CRF, then validates, prints and signs the form before sending it by fax;
- In case of failure to connect to the e-CRF, the investigator should complete, sign and send the SAE report form to the safety Department. As soon as the connection is restored, the investigator must complete the SAE report form in the e-CRF.

The investigator must comply with all requests for additional information from the sponsor.
For all questions relating to an adverse event report, the safety Department can be contacted via email at vigilance.drc@aphp.fr.

1.3 Role of the sponsor
The sponsor, represented by its safety Department, shall continuously assess the safety of each investigational medicinal product throughout the trial.

1.3.1 Analysis and declaration of serious adverse events

The sponsor assesses:
- the **seriousness** of all reported adverse events,
- the **causal relationship** between these adverse events and investigational medicinal product and any other treatments,

All serious adverse events for which the investigator and/or the sponsor suspect a causal relationship with the investigational medicinal product are classed as suspected serious adverse reactions.
- the **expectedness assessment** of the serious adverse reactions
Any serious adverse reaction whose nature, severity, frequency or outcome is inconsistent with the safety information described in the summary of product characteristics, or in the investigator's brochure if the product is not authorised, is considered unexpected.
The sponsor, acting through its safety Department, assesses the expectedness of the serious adverse reaction based on the information described below.

The sponsor will report all suspected unexpected serious adverse reactions (SUSARs), within the regulatory time frame, to the ANSM (French Health Products Safety Agency).
- The sponsor must send the initial report without delay upon receipt of the unexpected serious adverse reaction if it is fatal or life-threatening, or otherwise within 15 days from receipt of any other type of unexpected serious adverse reaction;
- The sponsor must provide all relevant additional information by sending follow-up reports, within 8 calendar days following receipt.

Any suspected unexpected serious adverse reaction must also be declared electronically using the Eudravigilance European adverse drug reactions database managed by the European Medicines Agency (EMA).
The sponsor must notify all the investigators about any information that could adversely affect the safety of the trial subjects.

1.3.2 Analysis and declaration of other safety data
This relates to any new safety data that may lead to a reassessment of the risk/benefit ratio of the trial or the investigational medicinal product, modifications in the investigational medicinal product use, the conduct of the clinical trial, or the clinical trial documents, or a suspension, interruption or modification of the protocol of the clinical trial or other similar trials.
The sponsor will inform the competent authority and the Ethics committee without delay after becoming aware of the emerging safety issue and, if applicable, describe which measures have been taken.
Following the initial declaration of emerging safety issue, the sponsor will declare to ANSM any additional relevant information about the new safety issues in the form of a follow-up report, which must be sent no later than 8 days after becoming aware of the information.

10.3.3 Annual safety report
The sponsor must prepare once yearly throughout the trial duration an annual safety report (Development Safety Update Report - DSUR) which includes, in particular:
- an analysis of safety data concerning trial subjects
- a description of the patients included in the trial (demographic profile etc.)
- a list of all the suspected serious adverse reactions that occurred during the period covered by the report,
- cumulative summary tabulation of all the serious adverse events that have occurred since the beginning of the clinical trial,
The report must be transmitted to ANSM no later than 60 days after the anniversary date corresponding to the date of authorization of the clinical trial by ANSM.
