# Supplementary material for: Effectiveness and Costs of Molecular Screening and Treatment for Bacterial Vaginosis to Prevent Preterm Birth: The AuTop Randomized Clinical Trial
Source: JAMA Pediatr. 2023 Jul 17;177(9):894–902. doi: 10.1001/jamapediatrics.2023.2250 (PMC10352927; doi:10.1001/jamapediatrics.2023.2250)
Supplement: Supplement 3. — eFigure. Schema of Treatment Algorithm eTable 1. Table of Primers and Probes Used in the Study eMethods 1. Molecular Analysis Procedure eMethods 2. European Patent eTable 2. Details of Cost Outcomes According to Randomly Assigned Groups in the AuTop Study eTable 3. Sensitivity Analyses With Complete Cases and With Missing Values Imputed Using the Worst-Case Scenario eTable 4. Characteristics of the Patients in the Subgroup of Nulliparous and Multiparous Women According to Randomly Assigned Group [file jamapediatr-e232250-s003.pdf]

Supplemental Online Content

Bretelle F, Loubière S, Desbriere R, et al; the Groupe de Recherche en Obstetrique et Gynécologie (GROG) Investigators. Effectiveness and costs of molecular screening and treatment for bacterial vaginosis to prevent preterm birth: the AuTop randomized clinical trial. *JAMA Pediatr*. Published online July 17, 2023. doi:10.1001/jamapediatrics.2023.2250

eFigure. Schema of Treatment Algorithm

eTable 1. Table of Primers and Probes Used in the Study

eMethods 1. Molecular Analysis Procedure

eMethods 2. European Patent

eTable 2. Details of Cost Outcomes According to Randomly Assigned Groups in the AuTop Study

eTable 3. Sensitivity Analyses With Complete Cases and With Missing Values Imputed Using the Worst-Case Scenario

eTable 4. Characteristics of the Patients in the Subgroup of Nulliparous and Multiparous Women According to Randomly Assigned Group

This supplementary material has been provided by the authors to give readers additional information about their work.

© 2023 Bretelle F et al. *JAMA Pediatrics*.

eFigure. Schema of Treatment Algorithm

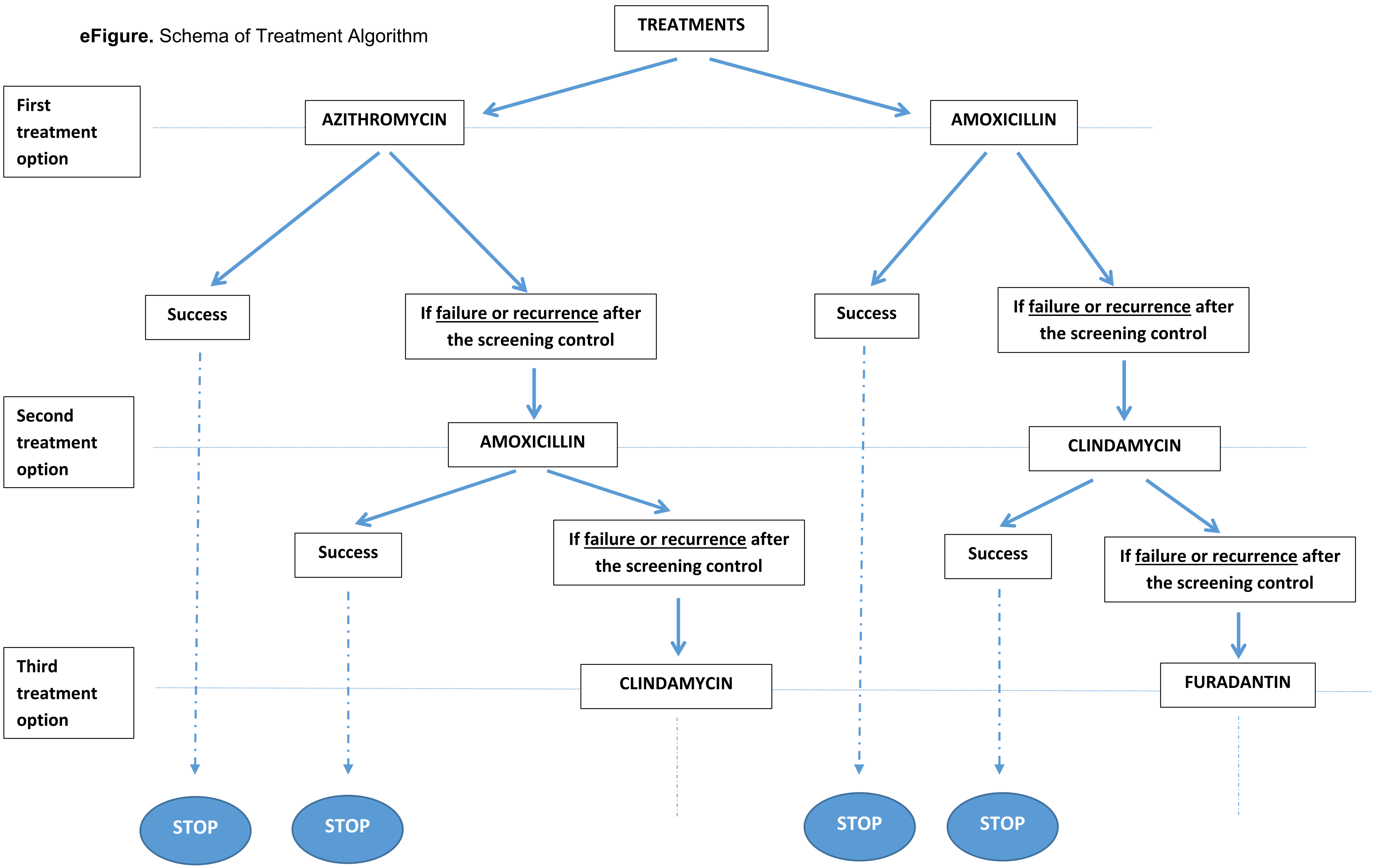

© 2023 Bretelle F et al. *JAMA Pediatrics*.

eTable 1. Table of Primers and Probes Used in the Study

PCR target	DNA target	Primers names	Sequences (5'→3')	Probes
Gardnerella vaginalis	*Cpn 60*	Gvaginalis_F	CGCATCTGCTAAGGATGTTG	VIC-TGCAACTATTTCTGCAGCAGATCCT
		Gvaginalis_R	CAGCAATCTTTTCGCCAACT	
Atopobium vaginae	16S rRNA	Atop_F	CCCTATCCGCTCCTGATACC	VIC-GCAGGCTTGAGTCTGGTAGGGGA
		Atop_R	CCAAATATCTGCGCATTTCA	
Human albumin	Exon 12	Alb_F	GCTGTCATCTCTTGTGGGCTGT	FAM-CCTGTCATGCCCACACAAATCTCTCC
		Alb_R	AAACTCATGGGAGCTGCTGGTTC	

PCR: polymerase chain reaction.

© 2023 Bretelle F et al. *JAMA Pediatrics*.

eMethods 1. Molecular Analysis Procedure

The vaginal specimens were discharged into 500 µl of BME (Basal Medium Eagle, Gibco) and 200 µl of suspension for DNA extraction using a QIAamp Tissue Kit (Qiagen, Courtaboeuf, France) on the BioRobot EZ1 automated system (Qiagen). Next, five µL of either a pure, undiluted DNA sample or a DNA sample diluted to 1/10µL was added to ready-to-use PCR mixes (see protocol study). Human albumin gene quantification was performed as an internal control to provide evidence for the presence of DNA and its quality. Master mixtures were systematically introduced as negative controls. qPCR was performed using a CFX96 Real-Time system (Bio-Rad Laboratories, Foster City, CA, USA).

© 2023 Bretelle F et al. *JAMA Pediatrics*.

eMethods 2. European Patent

(19) Europäisches Patentamt / European Patent Office / Office européen des brevets

(11) **EP 2 087 134 B1**

(12) **FASCICULE DE BREVET EUROPEEN**

(45) Date de publication et mention de la délivrance du brevet: **10.08.2011 Bulletin 2011/32**

(21) Numéro de dépôt: **07866534.6**

(22) Date de dépôt: **20.11.2007**

(51) Int Cl.: *C12Q 1/68* (2006.01)

(86) Numéro de dépôt international: **PCT/FR2007/052368**

(87) Numéro de publication internationale: **WO 2008/062136 (29.05.2008 Gazette 2008/22)**

(54) **METHODE DE DIAGNOSTIC ET DE SUIVI D'UNE VAGINOSE BACTERIENNE PAR QUANTIFICATION MOLECULAIRE**

VERFAHREN ZUR DIAGNOSE UND VERFOLGUNG VON BAKTERIELLER VAGINOSE MITTELS MOLEKULARER QUANTIFIZIERUNG

METHOD FOR DIAGNOSIS OF AND FOLLOWING A BACTERIAL VAGINOSIS BY MOLECULAR QUANTIFICATION

(84) Etats contractants désignés: **AT BE BG CH CY CZ DE DK EE ES FI FR GB GR HU IE IS IT LI LT LU LV MC MT NL PL PT RO SE SI SK TR**

(30) Priorité: **24.11.2006 FR 0610313**

(43) Date de publication de la demande: **12.08.2009 Bulletin 2009/33**

(73) Titulaire: **Université de la Méditerranée (Aix-Marseille II) 13284 Marseille Cédex 07 (FR)**

(72) Inventeurs:
- **BRETELLE, Florence 13014 Marseille (FR)**
- **FENOLLAR, Florence 13008 Marseille (FR)**
- **HENRY-MARY, Mireille 13850 Greasque (FR)**
- **MENARD, Jean-Pierre 13008 Marseille (FR)**
- **RAOULT, Didier 13008 Marseille (FR)**

(74) Mandataire: **Domange, Maxime et al Cabinet Beau de Lomenie 232, avenue du Prado 13295 Marseille Cedex 08 (FR)**

(56) Documents cités:
US-A1- 2005 095 603

- **BRADSHAW C S ET AL: "The association of Atopobium vaginae and Gardnerella vaginalis with bacterial vaginosis and recurrence after oral metronidazole therapy" JOURNAL OF INFECTIOUS DISEASES, CHICAGO, IL, US, vol. 194, no. 6, 15 septembre 2006 (2006-09-15), pages 828-836, XP009086643 ISSN: 0022-1899**
- **TABRIZI SEPEHR N ET AL: "Prevalence of Gardnerella vaginalis and Atopobium vaginae in virginal women" SEXUALLY TRANSMITTED DISEASES, LIPPINCOTT CO., PHILADELPHIA, US, vol. 33, no. 11, 1 novembre 2006 (2006-11-01), pages 663-665, XP009086642 ISSN: 0148-5717**
- **ZARIFFARD M REZA ET AL: "Detection of bacterial vaginosis-related organisms by real-time PCR for Lactobacilli, Gardnerella vaginalis and Mycoplasma hominis." 13 décembre 2002 (2002-12-13), FEMS IMMUNOLOGY AND MEDICAL MICROBIOLOGY 13 DEC 2002, VOL. 34, NR. 4, PAGE(S) 277 - 281 , XP002442546 ISSN: 0928-8244 page 278, colonne 1, alinéa 2 page 278, colonne 2, alinéas 2,3 tableau 1 le document en entier**
- **VERHELST RITA ET AL: "Comparison between Gram stain and culture for the characterization of vaginal microflora: Definition of a distinct grade that resembles grade I microflora and revised categorization of grade I microflora" BMC MICROBIOLOGY, BIOMED CENTRAL, LONDON, GB, vol. 5, no. 1, 14 octobre 2005 (2005-10-14), page 61, XP021002663 ISSN: 1471-2180**

Il est rappelé que: Dans un délai de neuf mois à compter de la publication de la mention de la délivrance du brevet européen au Bulletin européen des brevets, toute personne peut faire opposition à ce brevet auprès de l'Office européen des brevets, conformément au règlement d'exécution. L'opposition n'est réputée formée qu'après le paiement de la taxe d'opposition. (Art. 99(1) Convention sur le brevet européen).

EP 2 087 134 B1

Printed by Jouve, 75001 PARIS (FR)

(Cont. page suivante)

© 2023 Bretelle F et al. *JAMA Pediatrics*.

eTable 2. Details of Cost Outcomes According to Randomly Assigned Groups in the AuTop Study

	S&T (N=3,333) Mean [95%CI]	Control (N=3,338) Mean [95%CI]	p-value
**Intervention costs**			
Swabs, tests and treatment	**203.5 [196.5 – 209.5]**	**--**	
**Medical care costs**			
Mother	**2,750.4 [2,718.7 – 2,782.1]**	**2,762.9 [2,730.7 – 2,795.1]**	.587
Outpatient care during pregnancy	64.6 [61.5 – 67.7]	66.2 [63.2 – 69.3]	.241
Inpatient care during pregnancy	179.0 [156.3 – 201.7]	176.5 [154.1 – 198.9]	.515
Inpatient care at delivery/end of pregnancy	2,523.1 [2,506.5 – 2,539.6]	2,546.1 [2,528.7 – 2,563.3]	.088
Neonate £	**374.1 [296.8 – 451.4]**	**484.1 [371.8 – 596.5]**	.628
**Total Costs**			
Primary analysis population	3344.3 [3257.0 – 3431.0]	3272.9 [3149.5 – 3396.3]	.232
In nulliparous	3626.2 [3528.4 – 3723.9]	3727.7 [3519.5 – 3935.9]	.352
In multiparous	3061.0 [2918.2 – 3203.8]	2761.3 [2648.6 – 2874.0]	0.012

Mean [95%CI]: mean [95% Confidence Interval]· NICU: Neonatal Intensive Care Unit·
Pathological leaves were evaluated but for 62% of the population, the variable was not filled in and in the same proportion between the two groups, they were not valued·
£: Costs for neonates included NICU care, all transfers and home inpatient care·

© 2023 Bretelle F et al. *JAMA Pediatrics*.

eTable 3. Sensitivity Analyses With Complete Cases and With Missing Values Imputed Using the Worst-Case Scenario

Type of analysis	Variables/covariates	modality	N	aOR (95%CI)	P-value
**Analysis on complete cases (N=6,090)** [§]					
	Groups	S&T	3,041	0.61 (0.44, 0.83)	.002
	Tobacco	No	5,418	0.71 (0.51, 0.99)	.044
	Nulliparous	No	2,999	0.52 (0.37, 0.74)	<.001
	Caucasian	No	279	0.77 (0.47, 1.28)	.320
	Assisted pregnancy+	No	5,717	1.00 (0.81, 1.64)	.999
	Miscarriage	No	4,729	0.81 (0.62, 1.06)	.130
	BMI		6,090	1.00 (0.99, 1.00)	.540
	Age		6,090	1.01 (0.98, 1.03)	.900
	Groups S&T X Nulliparous No			2.08 (1.28, 3.37)	.003
**Analysis on imputed data using worse-case scenario (N=6,671)** [£]					
	Groups	S&T	3,333	0.87 (0.70, 1.07)	.170
	Tobacco	No	5,935	0.97 (0.75, 1.25)	.800
	Nulliparous	No	3,430	0.63 (0.49, 0.80)	<.001
	Caucasian	No	6,287	0.85 (0.61, 1.18)	.340
	Assisted pregnancy+	No	6,243	1.14 (0.80, 1.62)	.460

© 2023 Bretelle F et al. *JAMA Pediatrics*.

Miscarriage	No	5,180	0.83 (0.68, 1.01)	.055
BMI		6,671	1.00 (0.98, 1.01)	.910
Age		6,671	0.99 (0.98, 1.01)	.910

aOR: adjusted Odds Ratio; 95%CI: 95% Confidence Interval.
+: Assisted reproductive technology pregnancy
§: In the Complete cases analysis, the data set included all randomized individuals at baseline who completed the follow-up; for the primary medical outcome, N=6,371; considering missing data in covariates, N=6,090.
£: In the worst-case scenario, missing values were imputed by assigning the worst possible value of the outcome (i.e. failures, and putting the mean term for preterm birth) to dropouts in both groups; the interaction Groups X Nulliparous is not more significant.

© 2023 Bretelle F et al. *JAMA Pediatrics*.

eTable 4. Characteristics of the Patients in the Subgroup of Nulliparous and Multiparous Women According to Randomly Assigned Group

	**Nulliparous**		**Multiparous**	
**Characteristics**	**Screen & Treat (N=1,671)**	**Control (N=1,767)**	**Screen & Treat (N=1,662)**	**Control (N=1,571)**
Age (y)	29.1+/-4.9	29.3+/-5.0	31.9 (4.7)	32.2 (4.8)
Ethnic group – no. (%)[§]				
European	1,075 (64.7)	1,148 (65.6)	893 (54.0)	832 (53.4)
Caucasian*	70 (4.2)	68 (3.9)	77 (4.7)	76 (4.9)
North African	327 (19.7)	319 (18.2)	435 (26.3)	406 (26.0)
Sub-Saharan African	113 (6.8)	129 (7.4)	193 (11.7)	188 (12.1)
Asian	32 (1.9)	28 (1.6)	25 (1.5)	27 (1.7)
Other	45 (2.7)	59 (3.4)	32 (1.9)	30 (1.9)
Level > Bachelor's degree (yes) – no. (%)[§]	1,439 (86.6)	1,527 (86.8)	1,277 (77.5)	1,231 (79.2)
Active (yes) – no. (%)[§]	1,260 (75.9)	1,328 (75.5)	1,052 (63.6)	1,000 (64.1)
Previous early miscarriage (yes) – no. (%)[µ]	269 (16.2)	289 (16.5)	468 (28.2)	453 (28.9)
Previous abortion (yes) – no. (%)	313 (18.8)	319 (18.2)	320 (19.3)	326 (20.8)
Gestational age at inclusion (weeks)	15.6+/-2.8	15.7+/-2.8	15.5+/-2.8	15.4+/-2.8
Assisted reproductive technology pregnancy (yes) – no. (%)	121 (7.2)	154 (8.7)	68 (4.1)	49 (3.2)
Body mass index (kg/m^2)	23.4+/-4.5	23.7+/-4.6	24.7+/-5.2	24.7+/-4.9

© 2023 Bretelle F et al. *JAMA Pediatrics*.

Tobacco use (yes) – no. (%)§	183 (11.0)	215 (12.2)	181 (10.9)	146 (9.3)
Smoking during pregnancy (>10 cig/day) – no. (%)§	7 (0.4)	11 (0.6)	13 (0.8)	13 (0.8)
Alcohol use (yes) – no. (%)§	16 (1.0)	19 (1.1)	13 (0.8)	14 (0.9)
Vaginal toilet practice during pregnancy (yes) – no. (%)§	150 (9.1)	130 (7.5)	159 (9.7)	175 (11.4)

means +/- SD: mean +/- standard deviation.*: Native to the Caucasus region. §: These variables were self-reported. µ: Previous abortion includes therapeutic and spontaneous abortions

© 2023 Bretelle F et al. *JAMA Pediatrics*.
