# Supplementary material for: Effectiveness and Costs of Molecular Screening and Treatment for Bacterial Vaginosis to Prevent Preterm Birth: The AuTop Randomized Clinical Trial
Source: JAMA Pediatr. 2023 Jul 17;177(9):894–902. doi: 10.1001/jamapediatrics.2023.2250 (PMC10352927; doi:10.1001/jamapediatrics.2023.2250)
Supplement: Supplement 4. — Group Information. Groupe de Recherche en Obstetrique et Gynécologie (GROG) Investigators [file jamapediatr-e232250-s004.pdf]

Supplemental Online Content: Nonauthor Collaborators

*Indicates required information. Only first name, last name, and suffix will appear in PubMed.

*Group Name(s): Groupe de Recherche en Obstetrique et Gynécologie (GROG) Investigators							
***First Name and Middle Initial(s)**	***Last Name**	***Suffix (eg, Jr, III)**	Academic Degrees	Institution	Location (city, state/province, country)	Role or Contribution, eg, chair, principal investigator	Group (if more than 1 Group listed in the byline) and/or Subgroup (eg, Steering Committee)
Anne	Ego		MD PhD	CHU	Grenoble, France	Brainstorming group before conception	GROG
Catherine	Deneux-tharaux		MD PhD	Inserm	Paris, France	Brainstorming group before conception	GROG
Bruno	Carbonne		MD PhD	CHU	Monaco	Brainstorming group before conception	GROG
Camille	Leray		MD PhD	CHU	Paris, France	Brainstorming group before conception	GROG
Damien	Subtil		MD PhD	CHU	Lille, France	Brainstorming group before conception	GROG
Claude	D'Ercole		MD PhD	CHU	Marseille, France	Brainstorming group before conception	GROG
Denis	Gallot		MD PhD	CHU	Clermont Ferrand, France	Brainstorming group before conception	GROG
Christophe	Vayssiere		MD PhD	CHU	Toulouse, France	Brainstorming group before conception	GROG
Franck	Perrotin		MD PhD	CHU	Tours, France	Brainstorming group before conception	GROG
Francois	Goffinet		MD PhD	CHU	Paris, France	Brainstorming group before conception	GROG
Paul	Berveiller		MD PhD	CHU	Paris, France	Brainstorming group before conception	GROG
Loic	Sentilhes		MD PhD	CHU	Bordeaux, France	Brainstorming group before conception	GROG
Veronique	Debarge		MD PhD	CHU	Lille, France	Brainstorming group before conception	GROG
Laurent	Salomon		MD PhD	CHU	Paris, France	Brainstorming group before conception	GROG
Charles	Garabedian		MD PhD	CHU	Lille, France	Brainstorming group before conception	GROG

*Indicates required information. Only first name, last name, and suffix will appear in PubMed.

***First Name and Middle Initial(s)**	***Last Name**	***Suffix (eg, Jr, III)**	Academic Degrees	Institution	Location (city, state/province, country)	Role or Contribution, eg, chair, principal investigator	Group (if more than 1 Group listed in the byline) and/or Subgroup (eg, Steering Committee)
Jean Baptiste	Haumonté		MD PhD	CHU	Marseille, France	Brainstorming group before conception	GROG
Thibaud	Quibel		MD PhD	CHU	Paris, France	Brainstorming group before conception	GROG
Florent	Fuchs			CHU	Montpellier, France	Brainstorming group before conception	GROG
Karine	Baumstarck		MD PhD	AMU	Marseille, France	Brainstorming group before conception	steering Committee
Pascal	Auquier		MD PhD	AMU	Marseille, France	Brainstorming group before conception	steering Committee
Cécile	Fortanier		PhD	APHM CHU	Marseille, France	Brainstorming group before conception	steering Committee
