# Supplementary material for: Effectiveness and Costs of Molecular Screening and Treatment for Bacterial Vaginosis to Prevent Preterm Birth: The AuTop Randomized Clinical Trial
Source: JAMA Pediatr. 2023 Jul 17;177(9):894–902. doi: 10.1001/jamapediatrics.2023.2250 (PMC10352927; doi:10.1001/jamapediatrics.2023.2250)
Supplement: Supplement 5. — Data Sharing Statement [file jamapediatr-e232250-s005.pdf]

Data Sharing Statement

Bretelle. Effectiveness and Costs of Molecular Screening and Treatment for Bacterial Vaginosis to Prevent Preterm Birth. *JAMA Pediatr*. Published July 17, 2023. doi:10.1001/jamapediatrics.2023.2250

Data
Data available: Yes
Data types: Deidentified participant data
How to access data: on request at aap.drs@ap-hm.fr or sandrine.loubiere@univ-amu.fr
When available: With publication

Supporting Documents
Document types: None

Additional Information
Who can access the data: aap.drs@ap-hm.fr or sandrine.loubiere@univ-amu.fr
Types of analyses: the data will be available for any purpose after checking for the scientific interest of the purpose by the research teams and authors of the study
Mechanisms of data availability: the data will be available after approval of a proposal by authors and with a signed data access agreement
